# A Fuzzy-Set Qualitative Comparative Analysis of Potable Water Reuse Implementation Pathways

**DOI:** 10.1021/acs.est.5c18300

**Published:** 2026-04-03

**Authors:** Prakriti Sardana, Amy Javernick-Will, Sherri M. Cook

**Affiliations:** Department of Civil, Environmental, and Architectural Engineering, 1877 University of Colorado Boulder, Boulder, Colorado 80309, United States

**Keywords:** potable water reuse, implementation strategies, qualitative comparative analysis, workforce development, interagency agreements

## Abstract

The United States is facing increasing water scarcity challenges. Approaches that enable potable water reuse implementation are needed to increase supply and resilience. Therefore, we conducted a fuzzy-set qualitative comparative analysis after collecting data on 16 potable reuse projects using interviews, documentation, and field observations. We identified four generalized implementation approaches (i.e., success pathways); all shared the same causal conditions of committed interagency agreements, sufficient operator training, project leadership continuity, and positive media coverage. Three success pathways combined the shared causal conditions with low infrastructure integration burden, external public capital expenses funding, and one of multiple approaches for securing public acceptance: community spokespersons' local endorsement, a permanent demonstration/visitor center, or public education initiated 24 months before the implementation decision. The fourth pathway provides an implementation approach when infrastructure integration is complex and public funds are uncertain; it combined the shared causal conditions with internal/private capital expenses funding and public education initiated 24 months before the implementation decision. Further evaluating case knowledge also identified actionable guidance (e.g., onboard operators early to build confidence and earn certifications). This cross-case comparison’s strategies, which include multiple context-specific success pathways, can be applied to successfully implement potable water reuse and increase high-quality drinking water supplies.

## Graphical abstract

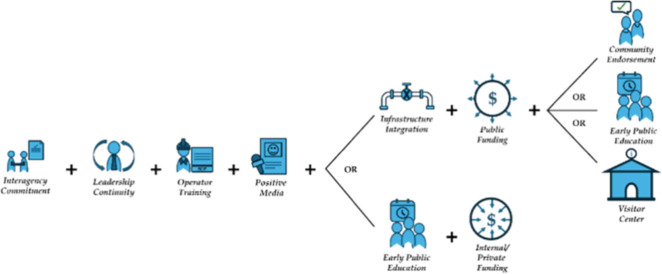

## 1 Introduction

Water scarcity is an increasingly global challenge driven by population growth, climate change, and overallocation of freshwater resources.^1–3^ In response, planned potable water reuse, which involves treating municipal wastewater to meet drinking water standards for indirect or direct augmentation, has emerged as a credible option worldwide to diversify water supply portfolios and improve long-term water system resilience. Successful examples in regions such as Australia,^4,5^ Singapore,^6,7^ and parts of the United States^8,9^ have demonstrated the technical feasibility of potable reuse, although implementation remains uneven due to governance, institutional, and public acceptance challenges.^10–12^ Building on this foundation, this study focuses on the United States as a region where we examine how combinations of conditions are associated with potable reuse implementation outcomes. While grounded in United States cases, the analysis generates insights that may inform utilities and decision-makers engaged in potable reuse efforts across a range of global contexts.

Previous research has helped utilities overcome many technical and institutional barriers to implementing potable reuse, such as from facility performance data,^9,13^ public health risk assessment frameworks,^14,15^ state regulatory guidelines,^16,17^ and public engagement strategies.^18,19^ However, given the range of contexts and complexity of reuse implementation, analyzing best practices or isolated factors in single cases has been insufficient to explain implementation success. For example, several case studies have been evaluated to richly describe how success was achieved in those specific projects.^8,20^ While this created a foundation of knowledge on implementation success, there is now a need for generalizing many successful implementation examples representing different contexts. Similarly, while multiple factors that may contribute to successful implementation have been evaluated,^11,12^ there is now a need to evaluate combinations of multiple factors instead of isolated factors, especially to better consider implementation in different contexts.

Further, the factors being evaluated need to be comprehensive and relevant to utility decisions. Existing research has evaluated combinations of structural, place-based factors, such as per-capita water availability, long-term rainfall, and population density.^21,22^ However, these factors may have limited relevance for utility decisions to determine whether projects move from concept to full-scale.^23,24^ Similarly, the literature frequently reduces multiagency agreements to simple cost-share arrangements between drinking water and wastewater agencies^25–27^ without discussing the scope and enforcement of interagency commitments. Evaluations of factors should be comprehensive, such that they can provide actionable guidance to inform and improve future implementation processes.

To better inform practice, there is a need for an expansive data set from diverse projects (i.e., cases) that allows for a systematic analysis of how different combinations of factors are linked to successful implementation. To this end, we conducted a systematic cross-case comparison of 16 potable water reuse projects in the United States by collecting comprehensive data using interviews, documentation, and field observations. To identify the combinations of factors associated with success, we conducted a fuzzy-set qualitative comparative analysis (fsQCA). Our findings advance theory on infrastructure implementation and provide actionable, evidence-based guidance grounded in contextual factors that can support utilities in transitioning potable reuse from a promising concept to full-scale implementation. Also, this analysis can offer actionable, context-sensitive guidance for utilities, municipalities, and consultants, including the identification of multiple strategies to successful implementation, to help them prioritize their limited resources and increase high-quality drinking water supply and resilience.

## 2 Methods

### 2.1 Case Selection and Data Collection

We identified candidate cases through multiple sources, including peer-reviewed literature, water sector reports, and conferences. Across these sources, we identified approximately 18 successful and 7 attempted potable reuse projects in the United States that had progressed beyond conceptual discussion to formal implementation activities between 2000 and 2025. Successful projects were defined as those that were operational for longer than one year or for which construction for full-scale operation had started. Attempted projects were defined as projects that had been formally pursued but were indefinitely delayed or canceled before proceeding to construction. For successful cases, we conducted desk research to prioritize cases that varied in climate and initiation year and represented diverse contexts across United States geographic regions, similar to our attempted cases. We contacted these cases with the goal of including 6–10 successful cases to align with the number of attempted cases. We evaluated the first nine cases that were able to engage in the case studies with this representation, and we had reached theoretical saturation, where new knowledge was not discovered after approximately five cases. Together, the 16 cases span 2.5 decades, climatic regions, and outcomes, providing a United States-based perspective on potable reuse implementation strategies (Table 1). To improve comparability across cases, we included only potable reuse projects that had progressed beyond purely conceptual discussion and had been formally pursued by the utility as a serious supply option, often through formal planning processes or alternative analyses in which reuse was assessed alongside other water supply options. Cases were included if utilities agreed to participate, and we were able to obtain primary and secondary data from each case using the same systematic protocol (Supporting Information Table S1) to ensure consistent and reliable^28,29^ data collection.^30^

**Table 1:** Case Characteristics^a^

case no.	climate	year of initiation	implementation outcome
1	arid	2002	successful
2	humid	2013	successful
3	humid	2014	successful
4	humid	2020	successful
5	arid	2016	successful
6	arid	2014	successful
7	arid	2013	successful
8	arid	2018	successful
9	humid	2014	successful
10	arid	2003	attempted
11	humid	2019	attempted
12	humid	2022	attempted
13	humid	2019	attempted
14	arid	2022	attempted
15	arid	2012	attempted
16	arid	2023	attempted

a The table displays climate, year of initiation, and implementation outcome for each case.

We collected data from October 2023 to July 2025. The primary methods of data collection included semistructured interviews and project documentation. We developed initial interview questions to collect data on an initial set of causal conditions (Supporting Information Section S1). The theory base we used to inform that initial set of causal conditions and underlying hypotheses was a previously published systematic literature review that identified factors influencing water reuse implementation.^10^ In this review, we created an affinity-grouped framework of factors and identified them as facilitators or barriers by analyzing published successful and attempted potable and nonpotable reuse case studies from global locations. Next, we piloted the initial interview questions in three cases. We found the questions to be effective and comprehensive in that they allowed conditions to appropriately emerge during the data collection and analysis phases, and so we proceeded with data collection for the remaining 13 cases. We conducted project overview interviews with utility project managers virtually. Then, while visiting each project site, we conducted multiple detailed interviews with utility project managers, operations and maintenance staff, and engineering consultants using three interview scripts that focused on potable reuse regulations, laws, and guidelines; stakeholder involvement and engagement; and funding, resources, and development (Supporting Information Section S2). In total, we conducted 60 interviews across the 16 cases (Table S2). During site visits, we also made unstructured field observations to document treatment technologies for cases that did not have publicly available data and to triangulate case evidence on permanent demonstration or visitor centers.

All interviews were audio-recorded and transcribed using Trint software v0.1.16.30. The project documentation analysis included project reports, funding documents, regulatory filings, and media articles. For example, we gathered documentation on bond ordinances and grant or loan agreements with dollar amounts and dates to identify percentages of external public capital expenses (CAPEX) funding. This documentation analysis allowed us to supplement and validate the interview responses. The research protocol was approved by the University of Colorado Institutional Review Board under Protocol 22-0554 and USEPA Human Studies Review Board HSR-001301.

### 2.2 Identification of Causal Conditions Using Thematic Analysis

We conducted qualitative coding using hybrid deductive–inductive thematic analysis, which combines theory- and data-driven approaches to identify, analyze, and report patterns within data.^31–33^ First, we imported all interview transcripts and documentation into the qualitative data analysis software NVivo v15.^34^ Next, we deductively developed an a priori codebook of the initial causal conditions. Then, we inductively modified those initial causal conditions and their initial operational definitions and coding, based on themes that emerged from the case data.

For example, we identified public education and awareness programs as an initial causal condition and initially defined it based on prior research as sustained, multichannel outreach designed to build public understanding and support for potable reuse.^35–37^ Then, to represent the three unique approaches implemented across all the cases, we updated it to three causal conditions: (i) having local endorsement from community spokespersons; (ii) initiating public education at least 24 months predecision (i.e., design-build award decision to implement potable reuse at full-scale); and (iii) having a permanent demonstration/visitor center. Additionally, we removed initial causal conditions that did not vary across cases (Supporting Information Section S3.1). For example, the initial causal condition of public decision-making participation had no cross-case variation, likely because it occurs primarily through statutory procedures (e.g., state/federal permitting notices and hearings).

The causal conditions (i.e., coding dictionary) (Table 2) were finalized once interpretative saturation was reached.^38^ Interpretative saturation refers to the point at which additional rounds of coding did not produce further modification of causal conditions or their operational definitions. Specifically, during the seventh coding cycle, there was no further modification of the causal conditions. First, we calibrated each causal condition by assigning set membership (i.e., fuzzy) scores (μ ∈ [0,1]) to reflect the extent to which each case exhibits each causal condition relative to other cases.^39^ Then, fuzzy scores were iteratively calibrated and theory-informed; we rescaled scores to reflect meaningful differences across cases through indirect calibration of qualitative data for eight causal conditions and direct calibration of quantitative data for two causal conditions. This resulted in our calibration guide (Supporting Information Tables S3–S10 and Figures S3 and S4). We also conducted sensitivity analyses using alternative anchor points (μ ±0.10) and a combined higher-order funding condition and found that they produced substantively similar calibration patterns and did not change the solutions. We generated the data matrix with the fuzzy score of each case’s causal conditions and outcome, assigning μ = 1 for each successful case (cases 1 to 9) and μ = 0 for each attempted case (cases 10 to 16). We analyzed calibration scores by condition for the mean, distribution, and standard deviation. Finally, we compiled detailed case study summaries for each case by qualitatively synthesizing all evidence related to each causal condition (Supporting Information Section S4).

**Table 2:** Causal Conditions Hypothesized to Influence Potable Reuse Implementation Success Based on Case Knowledge^a^

causal condition	summary of definition	citations
committed interagency agreements	utility executed, binding agreements among key agencies to assign roles, responsibilities, and resource commitments for the project.	^40–42^
continuity in project leadership	utility maintained stable, continuous management by a project lead or a core team of project engineers across project phases.	^43–45^
sufficient operator training	training enabled operators to competently run advanced treatment and monitor water quality and was documented and complied with certification requirements.	^11,46,47^
positive media coverage	media coverage of the project was predominantly positive in project framing, as reported by project participants.	^48–50^
low infrastructure integration burden	project included connections to existing treatment, conveyance, storage, and distribution systems, allowing for relatively shorter pipelines or fewer major new tie-ins.	^51–53^
public education ≥24 months predecision	proactive public education initiated at least two years before key governing decisions (i.e., full-scale implementation approval).	^54–56^
local endorsement from community spokespersons	public, on-record support affirming the reuse project’s safety and benefits from trusted local third-party spokespersons such as via letters, testimony, op-eds.	^8,54,57^
permanent demo/visitor center	a physical demonstration facility functioning as a visitor center was built to support public understanding and trust in safe potable water reuse.	^56,58,59^
sufficient external public CAPEX funding	adequate public funding from federal and/or state governments (via loans and/or grants) were documented and committed to cover project capital expenses.	^9,60,61^
sufficient internal/private CAPEX funding	adequate internal and/or private funding from utility cash and/or reserves, revenue bonds, and/or developer/private partner contributions were documented and committed to cover project capital expenses.	^62–64^

a The presence of causal conditions was hypothesized for success. Causal condition definitions were informed by the literature (see citations) and modified based on iterative case data analysis; the full definitions are in Supporting Information Section S3.2.

### 2.3 Identification of Success Pathways Using Fuzzy-Set Qualitative Comparative Analysis

We employed fsQCA to investigate how different combinations of causal conditions led to a successful implementation outcome. The fsQCA method uses case knowledge and set logic to support context-sensitive condition–outcome associations in small- to medium-N research.^21,65^ We imported the data matrix into the fs/QCA 4.1 software^66^ and systematically quantified set relations between the implementation outcome and all possible combinations of conditions (i.e., generated a truth table) to evaluate the extent to which a given combination of causal conditions was present when the implementation outcome was successful (Supporting Information Table S13). Using Boolean algebra and fuzzy logic, we minimized the truth table.^65,67^ We generated the final solution after specifying directional expectations (i.e., the presence of causal conditions was hypothesized for success; Supporting Information Section 4.3.1). This final solution identified “success pathways”, which are distinct combinations of causal conditions that were sufficient for successful potable reuse implementation.

We assessed the overall validity of the success pathways using two QCA metrics: solution consistency and coverage. Solution consistency is defined as the fraction of cases with a pathway that also had a successful implementation outcome (eq 1a). Solution coverage is defined as the fraction of cases with a successful implementation outcome that also had at least one pathway (eq 1b). We also calculated the necessity consistency and coverage of each causal condition and the success pathway. Necessity consistency (eq 2a) is defined as the fraction of cases with a successful implementation outcome that also had that causal condition. Necessity coverage (eq 2b) is defined as the fraction of cases with a causal condition that also had a successful implementation outcome. The consistency and coverage metrics reported in eqs 1a–2b follow established QCA definitions and usage.^67–69^

1a $$solution\;consistency=\frac{\Sigma \min\left(S_{i},O_{i}\right)}{\Sigma_{i}S_{i}}$$

1b $$solution\;raw\;coverage=\frac{\Sigma \min\left(S_{i},O_{i}\right)}{\Sigma_{i}O_{i}}$$

where *S*_*i*_ is case *i*’s set membership in the pathway solution and *O*_*i*_ is case *i*’s set membership in the successful implementation outcome.^70^

2a $$causal\;condition\;necessity\;consistency=\frac{\Sigma \min\left(C_{i},O_{i}\right)}{\Sigma_{i}O_{i}}$$

2b $$causal\;condition\;necessity\;coverage=\frac{\Sigma \min\left(C_{i},O_{i}\right)}{\Sigma_{i}C_{i}}$$

where *C*_*i*_ is case *i*’s set membership in a single causal condition and *O*_*i*_ is case *i*’s set membership in the successful implementation outcome.

## 3 Results and Discussion

### 3.1 Case Study Causal Conditions

When evaluating causal conditions individually, we found substantial variation (Figures 1 and 2). For example, successful cases 2, 3, 6, 8, and 9 had permanent demonstration/visitor centers, while successful cases 1, 4, 5, and 7 and all attempted cases did not. The timing of initiating public education before the first key design-build decision, which indicated the decision to implement potable reuse at full-scale, also varied from 120 months before to 12 months after the decision across successful and attempted cases (Figure 1). Since early education can shape political and organizational commitments to potable reuse acceptance,^71,72^ we calibrated public education timing based on the number of months before key design-build decision (Supporting Information Table S8).

**Figure 1:**
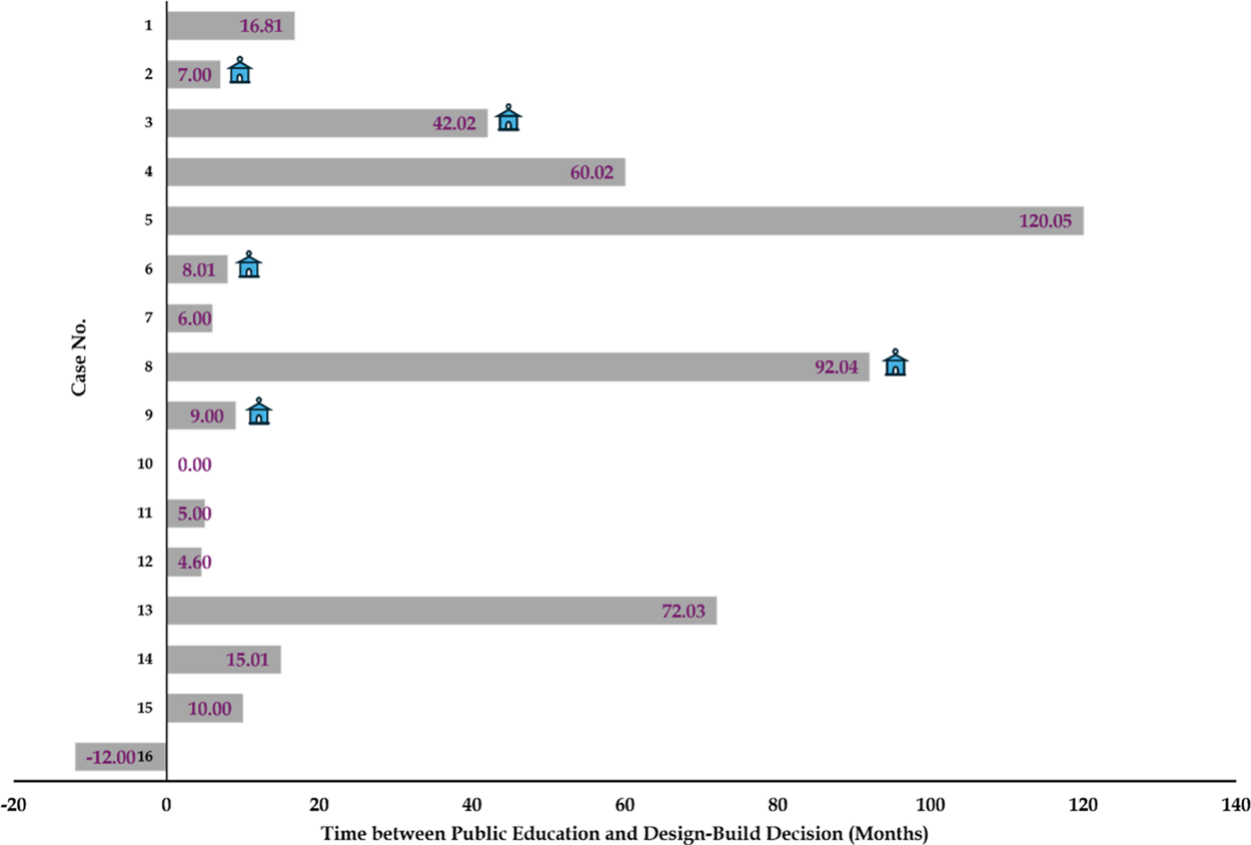
Timing of key public education milestones for 16 cases. Timeline of the first design-build decision, opening of a visitor or demonstration facility and start of a formal public education effort for the same cases, with each row representing one case. House symbols indicate cases with permanent demonstration/visitor centers.

**Figure 2:**
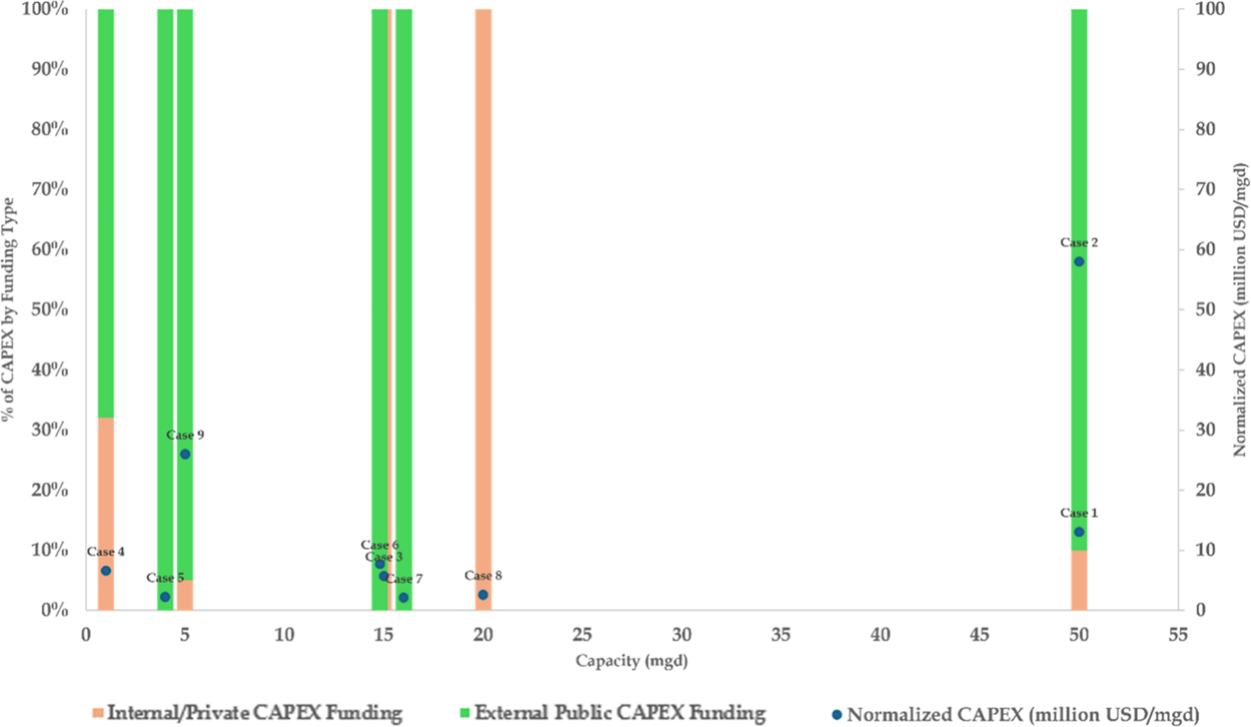
Distribution of normalized CAPEX by the funding source (external public or internal/private funding) and capacity (mgd) for successful cases (*n* = 9). The right *y*-axis shows normalized CAPEX (million USD/mgd) for each case. Blue markers show values of the normalized CAPEX for each case. Data labels next to markers show case numbers.

Another example was the variation of CAPEX normalized by capacity between the successful cases (Figure 2). For example, cases 1 and 2 are both 50 mgd projects, but their normalized CAPEX values are $13 million/mgd and $58 million/mgd, respectively. Also, case 9’s 5 mgd project with a normalized CAPEX of $26 million/mgd is more expensive than case 8’s $2.6 million/mgd value for its larger 20 mgd project. This causal condition could not be included, since it could not be calculated for all of the attempted cases.

Financial considerations were instead included by focusing on the financing approaches, which varied across the successful projects and could be calibrated across all cases. Specifically, some cases rely almost entirely on external grants and low-interest loans, whereas others are financed predominantly through internal or private sources. For example, cases 3, 6, 7, and 8 are similar sizes (14.8 to 20 mgd) and have similar normalized CAPEX values ($2.1 million/mgd to $7.7 million/mgd), but half of the cases entirely rely on external funding and the others on internal/private funding. To account for this heterogeneity, we have two causal conditions (sufficient internal/private CAPEX funding and sufficient external public CAPEX funding). We directly calibrated these by the percentage of total CAPEX by funding type (Figure 3).

**Figure 3:**
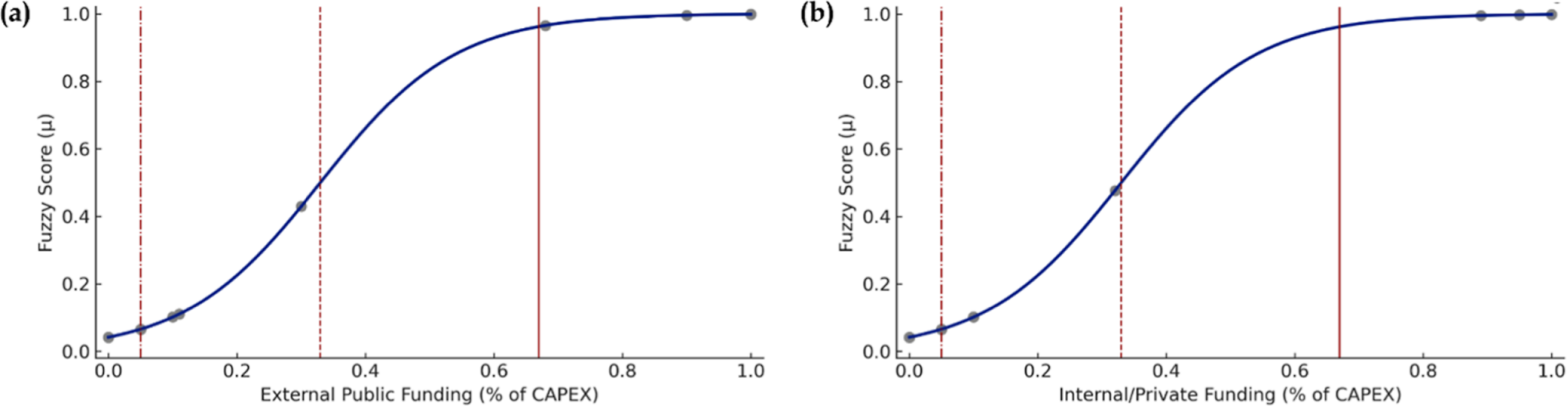
Direct calibration for (a) external public CAPEX funding and (b) internal/private CAPEX funding for successful cases (*n* = 9). The solid line represents the threshold for in-set membership; the dashed line represents the crossover point; the dashed-and-dotted line represents the threshold for out-of-set membership.

We also initially included project scale (i.e., capacity) and treatment technology causal conditions in the analysis, and both appeared in some intermediate solutions. However, their inclusion was not theoretically interpretable even though we observed variation under these conditions. Specifically, the scale of the project did not distinguish successful cases (Figure 2); cases ranged from small (1 mgd) to medium (15 mgd) to large (50 mgd) projects. The technology, which was either reverse osmosis-based or carbon-based advanced treatment trains, did not distinguish between successful and attempted projects, and case knowledge indicated that treatment train choice was often dictated by regulatory requirements for successful cases (e.g., cases 4, 6, 8, and 9). Therefore, we treated capacity and technology as domain causal conditions rather than conditions that differentiated cases’ implementation outcomes. Further discussion on removed causal conditions is provided in Supporting Information Section S3.1.

Calibrations of all of the final causal conditions show that several conditions tend to be either clearly present or clearly absent (Figure 4). For example, committed interagency agreements and continuity in project leadership are strongly in the set (μ > 0.5) of successful cases, whereas permanent demonstration/visitor centers and sufficient internal/private CAPEX funding are mostly out of the set (μ < 0.5). This distribution suggests that some organizational conditions are widespread among potable reuse projects, while others remain relatively rare. Comparing the mean membership scores of conditions across cases also reveals clear contrasts between successful and attempted projects. Successful cases have very high average membership for committed interagency agreements (mean μ = 0.96), continuity in project leadership (μ = 0.93), and sufficient operator training (μ = 0.83). In contrast, these causal conditions show much lower and more heterogeneous membership for attempted cases (μ = 0.52, 0.34, and 0.20, respectively).

**Figure 4:**
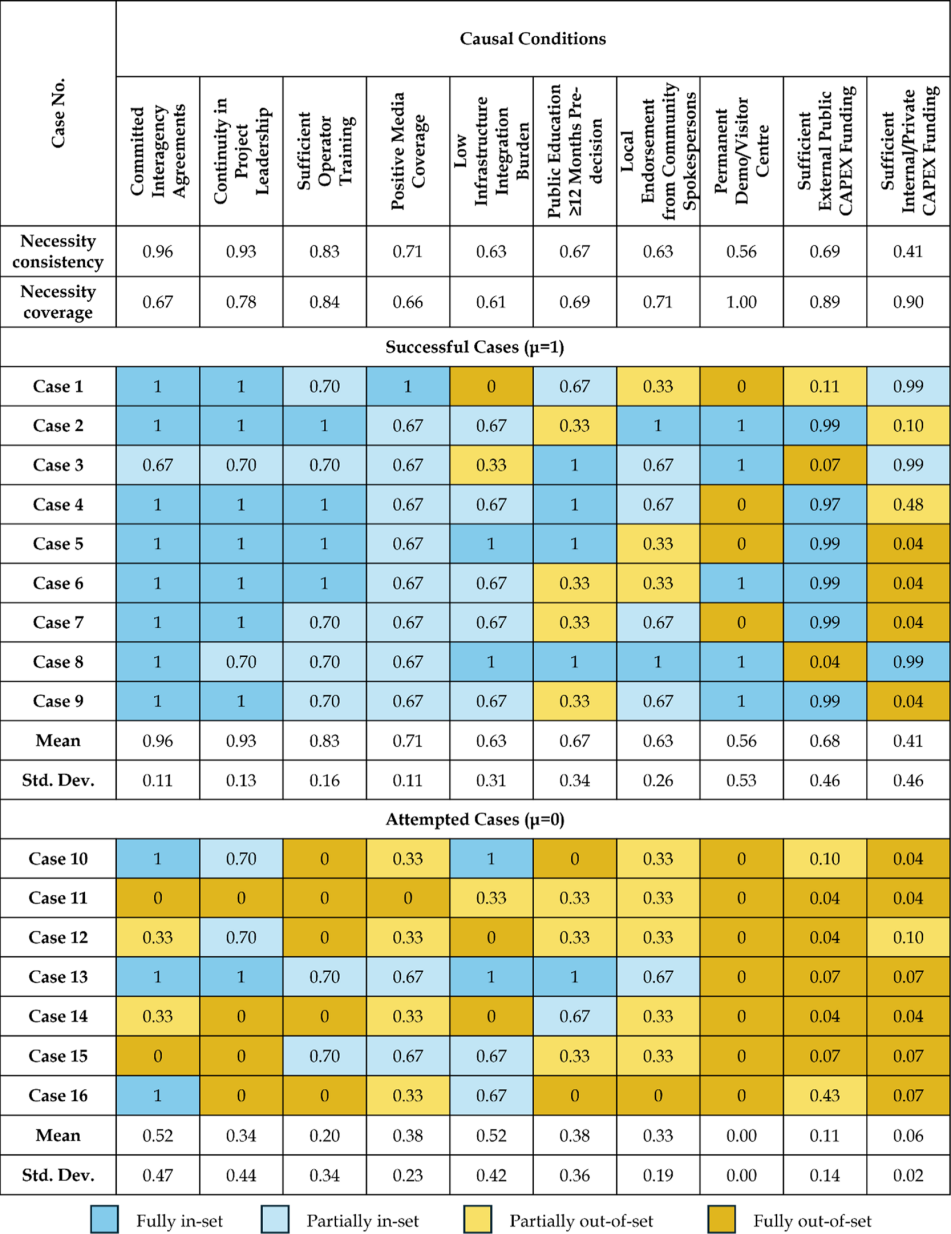
Data matrix with the fuzzy scores for each case’s outcome and causal condition. The last two columns show descriptive statistics (i.e., mean and standard deviation) for each causal condition. Necessity consistency and coverage of the presence of individual causal conditions are displayed below each condition; necessity consistency and coverage of the absence of individual causal conditions are further displayed in Supporting Information Table S12. Supporting Information Figure S1 shows scatter plots of each causal condition’s distribution of calibrated membership scores for all 16 cases.

Conditions related to positive media coverage and public acceptance approaches (i.e., early public education, local endorsements, visitor centers) also tend to have higher memberships among successful projects (μ > 0.5) than among attempted projects (μ < 0.5). These descriptive patterns highlight that institutional and organizational conditions, rather than funding alone, differentiate successful from attempted implementation outcomes. Finally, conditions with higher within-set variation provide insight into the configurational analysis because they help distinguish among otherwise similar projects. Thus, the calibrated data matrix serves both as a descriptive summary of how strongly each project exhibits each condition and as the empirical basis for constructing the fsQCA truth table (Supporting Information Table S13). Overall, we found that while certain conditions may be close to necessary for success, none is sufficient in isolation, motivating the examination of combinations of conditions using fsQCA.

### 3.2 Pathway Results

We identified four success pathways that display unique combinations of causal conditions (i.e., alternative strategies) that were sufficient to achieve successful potable reuse implementation (Figure 5). Success pathways are organized by shared causal conditions not chronology, and branches indicate alternative strategies used by different successful cases. All success pathways achieved high overall solution consistency (*n* = 0.99) because cases exhibiting these combinations of conditions consistently resulted in success and high overall solution coverage (*n* = 0.67) because all successful cases aligned with at least one pathway.

**Figure 5:**
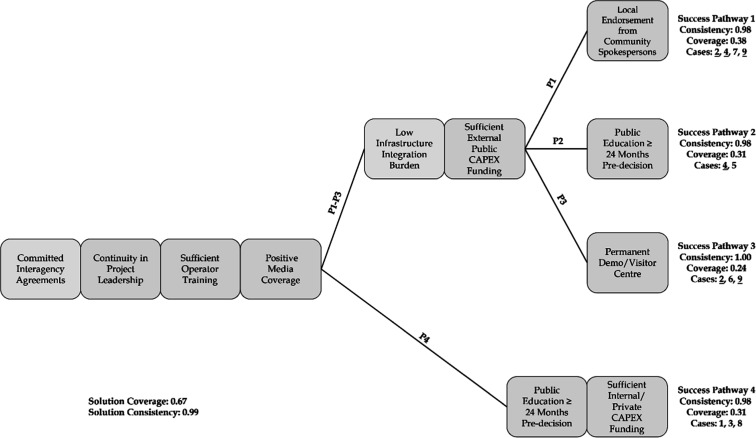
Four success pathways (labeled P1 to P4) that show the combination of causal conditions associated with successful potable reuse implementation (solution consistency = 0.99, solution coverage = 0.67). Quantitative pathway metrics showing each pathway’s raw coverage and consistency values and cases covered are shown next to each pathway. There are four shared conditions before the pathways branch. Conditions are ordered from left to right by their common presence in the pathways, followed by necessity scores. Pathways are arranged from top to bottom based on pathway coverage scores and by shared conditions rather than chronology. Cases with the conditions on each path are displayed next to the pathways they followed. Underlined case numbers (2, 4, and 9) show cases that followed multiple pathways (i.e., they had all conditions present in the associated pathways).

All success pathways (P1–P4) shared the same four causal conditions of committed interagency agreements, sufficient operator training, continuity in project leadership, and positive media coverage. Three of the four success pathways (P1–P3) combined the four shared causal conditions with low infrastructure integration burden, sufficient external public CAPEX funding, and one of multiple approaches for securing public acceptance: having local endorsement from community spokespersons (P1), initiating public education at least 24 months before the decision to implement potable reuse at full-scale (P2), or having a permanent demonstration/visitor center (P3). The fourth success pathway (P4) required a combination of the four shared causal conditions, with initiating public education at least 24 months before decision and having sufficient internal or private CAPEX funding. Overall, P1 reads as committed interagency agreements AND continuity in project leadership AND sufficient operator training AND positive media coverage AND low infrastructure integration burden AND sufficient external public CAPEX funding AND local endorsement from community spokespersons.

We assessed the robustness of our results by varying the sensitivity of the consistency threshold used for the truth table generation. In addition to the main analysis, which used a consistency cutoff of 0.80, we re-estimated the truth table and solutions using more permissive and restrictive thresholds of 0.70 and 0.90, respectively. In both cases, the same configurations were retained in the truth table, yielded the same prime implicants, and produced identical intermediate solutions and case coverage. As a result, we determined that the solution pathways are insensitive to reasonable changes in the thresholds used to construct the truth table. While these pathways capture the breadth of decision-making complexity in potable reuse, it is also important to inform practice and knowledge sharing by evaluating the specific challenges encountered during the cases’ implementation processes and how cases navigated any challenges successfully.

Successful cases used several approaches that helped them establish different types of committed interagency agreements, including (i) delivery and purchase agreements and (ii) joint regulatory agreements. Cases used these agreements to precommit partners and address the authority, finance, operations, and permitting issues that can derail implementation. Since these issues often emerge at crucial and unexpected times (e.g., during permit-mandated public comment periods, financing milestones, service disruption due to drought), it can be helpful to evaluate the types of issues that cases needed to navigate. For example, delivery/purchase agreements detailed water purchase commitments between the implementing utility and regional stakeholders to finalize financing and operations arrangements. The utility in case 8 established explicit delivery-volume and purchase-exchange commitments with private developers. The developers committed to paying capital costs toward the utility’s potable reuse system expansion and cover operation and maintenance costs for the water delivery system; the utility committed to deliver a specified quality and quantity of reuse water to the private developers with topmost priority. Joint regulatory agreements detailed coordinated permitting steps and clearly assigned agency responsibilities to address authority, operations, and permitting issues (cases 2, 3, and 6). For example, the utility in case 6 created a joint regulatory work program that committed regional watershed and flood-control agencies to provide access to augmentation points and conveyance easements and committed wastewater facilities to provide secondary-effluent volume inflows and shared monitoring and reporting.

Cases 1 and 9 used both types of agreements in project planning to address permitting, operations, and financing issues. In case 1, the utility negotiated expansion of its conveyance system across multiple jurisdictions via joint regulatory workplans and created a regional partnership to sell surplus water supplies in high-flow years via delivery/purchase contracts. In cases 4, 5, and 7, the utilities did not require an interagency agreement and only needed city council approval, meaning that each of these utilities had all the responsibility and power over project-related decisions. Even though our findings show that these agreements are central to implementation success, committed interagency agreements are under-examined in the potable reuse literature, aside from a few practitioner guidance documents.^27,40^ We recommend future research systematically compare which clause-level (e.g., rights-of-way, credit-trading mechanics) and project-phase-level evaluations of which provisions prevent project permitting delays and cost overruns.

To achieve sufficient operator training, successful cases used several approaches. First, early hiring and engagement of operators was repeatedly cited by utility managers as a success factor. For example, the utilities in cases 1 and 7 hired operators early in the project implementation process to help cultivate ownership, accountability, and site-specific institutional knowledge, especially through construction observation. Other successful cases (cases 2, 3, 5, 6, and 8) used pilot plants or demonstration facilities to help train operators through hands-on experience, which allowed operators to gain required hours of experience and competencies needed to satisfy state certification requirements, thus mitigating regulatory compliance barriers to implementing potable reuse projects. For example, the utilities in cases 3 and 8 created new job classifications and training plans by using their demonstration facilities. They included training modules for local high school and community college students to recruit new operators and for existing operators to promote advanced training. Across cases, operators helped convert advanced treatment knowledge from procedure manuals into routinized practice for full-scale implementation (e.g., refining set points and maintenance intervals, proactively testing incident responses). Overall, we found two practical approaches that cases used to achieve sufficient operator training: hiring early and providing hands-on experience. These approaches can help future potable reuse projects achieve specialized operator training beyond standard certifications, which previous research has stated as a requirement for success.^11,73^

The successful cases’ focus on workforce development and stability also included an emphasis on sustaining continuity in project leadership, by having either a dedicated project lead (e.g., project manager, utility director) or a dedicated team of decision-making engineers. For example, the utilities in cases 1, 5, 7, 8, and 9 had utility directors and general managers that were long-tenured internal water reuse champions and served as project leads across planning, piloting, and early project operations. They provided strategic project direction and consistent decision-making despite externally changing expectations (e.g., permitting requirements). They also served as the sole trusted channel that delivered monthly project updates to the public, funders, and regulators. The utilities in cases 2, 3, 4, and 6 maintained continuity in project leadership through a dedicated team of decision-making engineers. These teams bridged project phases, established full-scale planning and design goals, ensured consistent technical vision, and helped offset any disruption caused by the departure of project leads. This finding is consistent with project management research, which posits that continuity of project leadership, via a single champion or a principal program team, helps preserve strategic direction and maintains knowledge continuity for a project.^43,74,75^ Our analysis also showed that continuity in project leadership via the presence of a dedicated project lead or team helped maintain a consistent project narrative for the public, funders, regulators, and media.

To achieve predominantly positive media coverage, cases reported the use of utility-preferred branding (Figure 6), which was reinforced by transparent public information channels and, if possible, by a consistent project lead. The messaging in all of these cases emphasized the safety of the water, highlighting that the purified water would be blended into a natural water source to ease public water quality concerns. For example, the utilities in cases 1 and 4 chose consumer-friendly branding terms such as “purified” and “natural” water. The utilities in cases 2, 3, 4, 6, 8, and 9 pressure-tested content by soliciting feedback from focus groups and public advisory committees with citizens’ juries on branding potable reuse water as “Pure Water” before deciding on broad public education branding. Further, the utilities in cases 1, 4, 5, 6, 7, and 8 reinforced public credibility by branding their project as state-led drought-security and water independence initiatives.

**Figure 6:**
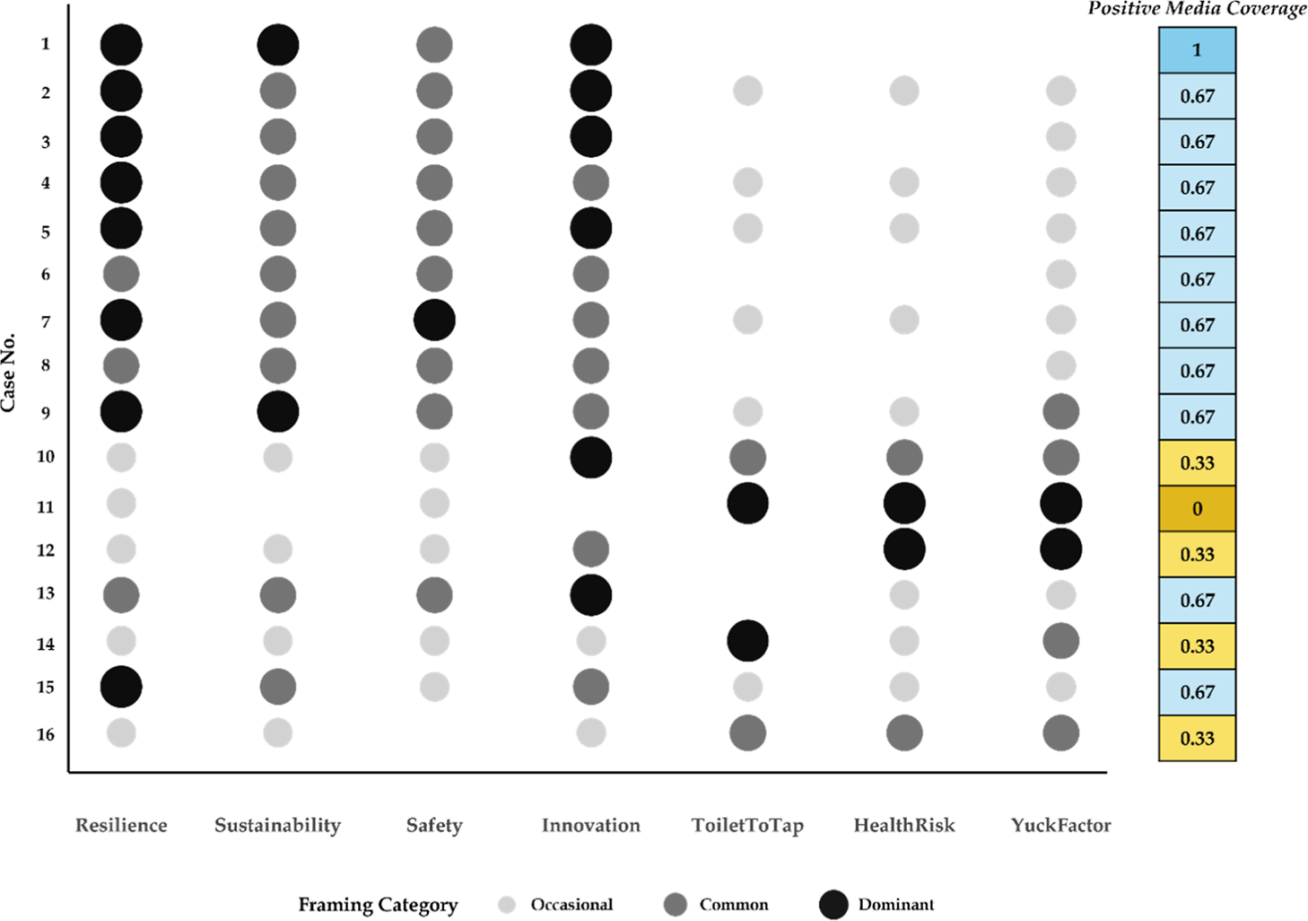
Media framing categories for successful (cases 1 to 9) and attempted (cases 10 to 16) cases. Bubble color and size indicate the coded prevalence of each framing category in local media coverage. Blank spaces indicate “absent” framing category. The colored column shows each case’s calibration fuzzy score for the positive media coverage causal condition.

All successful cases reinforced their branding efforts through transparent communication with the public. Specifically, all cases created a utility-controlled Web site about their project with a frequently asked questions section, maintained an active social media presence (e.g., regular project updates on Facebook, producing YouTube videos), and engaged with the media through local TV or radio briefings. Most successful cases also conducted public tours of the potable reuse plant (cases 1, 2, 5, 6, 7, 8, and 9) and/or had real-time water-quality dashboards that enabled two-way feedback to address public health concerns via transparent public information channels (cases 2, 3, 5, 6, 7, and 8), which helped reinforce the projects’ positive media coverage. These findings emphasize the importance of utilities proactively taking the lead in shaping media narratives, which can strongly influence public acceptance^50,76^ and have traditionally stigmatized potable reuse (e.g., “toilet-to-tap”).^77,78^

While it is valuable to see unique approaches to individual causal conditions, it is also valuable to see the interconnectivity between causal conditions by examining the entire pathway and its combination of conditions. For example, we found different combinations of conditions associated with external public funding and internal/private funding, showcasing that successful potable reuse implementation did not depend on a single financing model and suggesting that different conditions may need to be strategically considered based upon the financial model used. Specifically, the first three success pathways (P1 to P3) benefited from the connection between low infrastructure integration burden and sufficient external public CAPEX funding and one of multiple public acceptance strategies: local endorsement from community spokespersons (P1), public education initiated at least 24 months predecision (P2), or a permanent demonstration/visitor center (P3). For example, case 4 was primarily grant-funded with 68% of CAPEX covered by grants that did not need to be repaid; this case’s lower infrastructure integration burden made it feasible to cover the remainder of the CAPEX through internal utility enterprise funds. In contrast, the fourth pathway (P4) combined sufficient internal/private CAPEX funding with early public education. For example, in cases 1 and 3, utility boards allocated internal funding through enterprise funds and municipal bonds, and the utilities used early, evidence-first public education to build familiarity and acceptance. Together, these findings suggest that different financial models may require different complementary organizational and public engagement strategies.

All three pathways (P1–P3) secured public acceptance using one of multiple approaches. Success pathway P1 (cases 2, 4, 7, and 9) used local endorsement from community spokespersons (e.g., local business partnerships, environmental conservancy groups, and academic and public health organizations) that helped translate utilities’ water quality claims into relatable experiences. For example, case 2 achieved endorsements by leveraging long-standing, informal utility networks via small local business partnerships (e.g., hotels, breweries). Cases in this pathway varied in starting conditions: some entered the process in a more exploratory but formal planning mode, whereas others began with stronger institutional backing and a clearer mandate to improve water resilience. For example, cases 2 and 9 operate in humid regions and were initially motivated by wastewater discharge regulations and long-term supply resiliency, whereas cases 4 and 7 pursued potable reuse in response to severe droughts and strong political pressure for water security (SI Section S4). Despite this heterogeneity in starting conditions, these cases shared the same combination of conditions associated with success, suggesting that the identified pathway was not limited to utilities with a single type of initial motivation or baseline level of commitment. For example, across cases, continuity in project leadership was important for sustaining momentum, maintaining institutional memory, and carrying the project from early exploration into full-scale implementation. Success pathway P2 (cases 4 and 5) secured public acceptance by initiating public education at least 24 months predecision to implement potable reuse at full-scale (Figure 1). For example, case 5’s utility initiated formal public education ten years before the design-bid-build award decision, providing regular opportunities for the public to visit the wastewater treatment plant. Further, the utility built a pilot to host public and media visits to achieve positive media coverage six months before regulatory permitting full-scale implementation was secured. The utility emphasized early, focused updates including routine briefings to a standing citizens’ advisory committee instead of a broad education program by branding the project as a long-term drought-security initiative, allowing the project to proceed to full-scale implementation without vocal public resistance.

Success pathway P3 (cases 2, 6, and 9) secured public acceptance using the third approach: a permanent demonstration/visitor center. Across these cases, utilities built permanent demonstration/visitor centers that included museum-grade education centers and hosted water tastings to normalize the concept of potable reuse for the public. These cases also maintained an in-house communications team that managed the center, led sustained public education activities, and served as liaisons among partner agencies to support the development of committed interagency agreements. Overall, although existing scholarship recommends expansive and resource-intensive outreach,^79^ our analysis suggests that public acceptance could be achieved by one of these three context-specific approaches that address specific public acceptance barriers.

Most cases used a combination of these approaches in addition to achieving positive media coverage. For example, cases 2 and 9 invested in permanent demonstration/visitor centers and obtained local endorsement from trusted community spokespersons to secure public acceptance. Case 4 had local endorsement from community spokespersons and initiated early education. However, these three cases were represented by multiple pathways (case 2 and 9 by P1 and P3 and case 4 by P1 and P2), indicating that each associated pathway was equally sufficient for that case’s success. Since each pathway includes different approaches to securing public acceptance, it might be possible for a future project to implement only one of these approaches instead of multiple. The timing and sequencing of these approaches may also have been decisive in securing public acceptance, so future research should conduct detailed timeline analyses to evaluate when specific approaches become critical to projects’ success.

Success pathway P4 (cases 1, 3, and 8) combined sufficient internal (e.g., via utility enterprise funds, municipal bonds, cash reserves) or private (e.g., via developer or industrial funding) CAPEX funding and initiating public education at least 24 months predecision. For example, in cases 1 and 3, the utilities’ boards of directors decided to allocate sufficient internal CAPEX funding to the projects. Enabled by continuity in project leadership, early timing of their evidence-first public education efforts allowed the utilities to use piloting data to answer anticipated questions about topics such as the potable reuse treatment train’s ability to remove contaminants. These cases’ education approach built public familiarity with potable reuse to help secure public acceptance early and enabled them to be entirely internally funded via enterprise funds and municipal bonds.

Overall, our analysis identified four empirically grounded pathways through which potable water reuse projects have been successfully implemented. These pathways provide utilities and planners with a practical framework to employ on the basis of their situated, contextual projects rather than a prescriptive set of best practices. Utilities embarking on a new potable reuse project can use the pathways by comparing their own institutional, financial, operational, and public engagement conditions to those associated with each pathway identified in this analysis. Specifically, utilities can evaluate the presence or absence of the causal conditions examined in this study (e.g., interagency agreements, operator training capacity, financing strategy, and public engagement approach) and evaluate how closely their current project configuration aligns with one or more pathways to guide future decisions for targeted interventions that may be needed to support project progression. If critical conditions associated with a pathway are absent, we recommend that utilities prioritize interventions to address these gaps. For example, a utility with limited access to external funding may consider strategies aligned with the pathway characterized by internal funding combined with early public education to strengthen the internal project commitment. Similarly, based on our results, if leadership turnover has delayed alignment on project scope, permitting, or funding, then ensuring continuity in project leadership should be prioritized by designating a single accountable lead with a succession plan or a small core team with clear decision-making rights across phases. In cases where projects stall due to absent or ambiguous interagency commitments, the pathways point to the importance of formalizing agreements with specified responsibilities, permitting steps, timelines, quantity and quality commitments, and data-sharing expectations to move the project forward. The guidance from our analysis can provide utilities, municipalities, and consultants with clear strategies so that a potable reuse project makes progress to full-scale implementation to ensure high-quality drinking water supply and resilience. In this way, the identified pathways support utilities in identifying context-sensitive strategies for advancing potable reuse projects toward implementation.

Our findings extend prior potable reuse research that has often examined implementation factors in isolation. We also aimed to identify actionable utility-level factors associated with implementation outcomes. Although several causal conditions, such as public education efforts, media coverage, funding availability, and interagency coordination, may appear to reflect a broader construct of institutional capacity, we treated them as analytically distinct because both our prior systematic literature review and our case data indicated that they operate in different implementation domains, involve different actors, and present different intervention points for utilities. As shown in our case evidence, for example, public education timing varied widely across cases (i.e., from 120 months before to 12 months after the decision), while interagency agreements involved separate processes, such as delivery/purchase contracts or joint regulatory workplans. Future research could nevertheless explore whether aggregating these dimensions into a higher-order institutional capacity condition alters the resulting pathways and thereby is more useful for policy-level recommendations.

Finally, our results show that successful implementation depends on how these causal conditions combine. The recurrence of committed interagency agreements, sufficient operator training, continuity in project leadership, and positive media coverage across all pathways suggests that potable reuse implementation is not solely a technical challenge but also one of institutional capacity and sustained public communication.^18,80^ This finding complements broader water governance and infrastructure transition scholarship, which similarly emphasizes the importance of interagency alignment, leadership stability, and meaningful public engagement.^54,81^ Overall, by identifying multiple equifinal pathways, our analysis also helps to explain why successful projects may rely on different financing arrangements and public acceptance strategies while still achieving successful implementation.

## Supplementary Material



## Notes

(Section S1) Data collection protocol; (Section S2) interview scripts and overview of semistructured interviews conducted by case study; (Section S3) extended fsQCA analytical procedure, including preliminary minimization and removal of causal conditions, detailed definitions and calibrations of causal conditions, and step-by-step QCA procedure using fs/QCA 4.1 software; and (Section S4) case summaries of all included case studies (PDF)
