# Supplementary material for: A Fuzzy-Set Qualitative Comparative Analysis of Potable Water Reuse Implementation Pathways
Source: Environ Sci Technol. 2026 Apr 3;60(14):10704–16. doi: 10.1021/acs.est.5c18300 (PMC13085517; doi:10.1021/acs.est.5c18300)
Supplement: Supplementary file 1 [file es5c18300_si_001.pdf]

Supporting Information

for

A Fuzzy-Set Qualitative Comparative Analysis of Potable Water Reuse Implementation Pathways

Prakriti Sardana;[a] Amy Javernick-Will;[a] Sherri M. Cook[a*]

[a]Department of Civil, Environmental, and Architectural Engineering, University of Colorado Boulder, Boulder, CO 80309

[*]Corresponding author: Email: sherri.cook@colorado.edu, Phone: 303-735-7288, Fax: 303-492-7317, Address: 4001 Discovery Drive, 607 UCB, Boulder CO 80309

Number of pages: 65

Number of tables: 13

Number of figures: 4

Table of Contents

S1. Data Collection Protocol 4
S2. Interview Scripts 5
Interview 1: Project Overview: Potable Reuse at Your Utility 5
Interview 2: Stakeholder Involvement and Engagement 7
Interview 3: Potable Reuse Regulations, Laws and Guidelines 9
Interview 4: Funding and Resources & Development 11
S3. Extended fsQCA Analytical Procedure 13
S3.1. Preliminary Minimization and Removal of Causal Conditions 13
S3.2. Detailed Definitions and Calibrations of Causal Conditions 18
Committed Interagency Agreements 18
Continuity in Project Leadership 20
Sufficient Operator Training 21
Positive Media Coverage 22
Low Infrastructure Integration Burden 24
Public Education ≥ 24 Months Pre-decision 25
Local Endorsement from Community Spokespersons 27
Permanent Demo/Visitor Center 28
Sufficient External Public CAPEX Funding 29
Sufficient Internal/Private CAPEX Funding 32
S4.3. Step-by-step QCA procedure using fs/QCA 4.1 software 34
S4.3.1. Process 34
S4.3.2. Subset/Superset Analysis 41
S4. Case Summaries 45
Case 1 49
Case 2 50
Case 3 51
Case 4 52
Case 5 53
Case 6 54
Case 7 55
Case 8 56
Case 9 57
Case 10 58
Case 11 59

Case 12 60
Case 13 61
Case 14 62
Case 15 63
Case 16 64
References 66

S1. Data Collection Protocol

Typically, for case-based research, we seek 6 sources of evidence to corroborate information and insight into the case with as many varied sources as feasible.[1] Table S1 enlists each type of data, data sources and specific information that we collected. Coding and analysis for each case study was based on triangulated information from all relevant data sources. For this work, *participant observation* and *physical artifacts* were irrelevant data sources.

Table S1. Data Collection Protocol.

Type of Data	Data Source	Specific Information
**Project Documentation**	Project website, if any	Information such as plant capacity, service population, treatment processes.
	Media coverage of the project	To gauge predominant media sentiment (positive/negative)
	Project proposal and economic feasibility reports	To identify %s of CAPEX from various funding sources
	Prior case study reports, if any	To identify and validate any strategies, facilitators, barriers and lessons learned mentioned
	Relevant regulatory policy documents	To identify state regulatory precedent for potable reuse
**Archival Records**	U.S. Drought Monitor	To identify cumulative and average drought periods
	Census data	To identify public water consumer demographic factors
**Interviews**	#1: Project overview interview	
	#2: Stakeholder involvement & engagement	
	#3: Potable reuse policy, laws & guidelines	
	#4: Funding and resources & development	
**Direct Observation**	Site visit to each utility	Informal conversations with utility managers and operators; photographs and videos of the potable reuse facility, primarily for dissemination and overview of state of existing infrastructure

S2. Interview Scripts

Interview 1: Project Overview: Potable Reuse at Your Utility

(60 minutes)

The results of this interview will help us assess water reuse barriers and facilitators and identify pathways to successful water reuse implementation.

Questions:

1. Please describe your role in the water reuse project at your utility, including your title and main responsibilities (past and present).
2. Please briefly describe the water reuse project operation.
    a. What is the daily average flow rate?
    b. What is the source water?
    c. What are the intended end uses of the treated water?
    d. What treatment trains and technologies are in operation?
    e. Who are the intended water consumers?
    f. What are the expected benefits of this project for the community?
    g. Was a cost-benefit analysis performed?
        i. What were the environmental, social, and economic impacts considered?
2. Please describe the main activities performed and decisions made during the **initiation** phase, which entails project conception.
    a. When was the project initiated (year/month)?
    b. What were the main reasons for pursuing potable reuse at your utility?
    c. What were the main activities performed and major decisions made during this phase?
        i. Why were these decisions made during this phase?
        ii. Who were the key players in making major decisions during this phase?
    d. What were the main challenges faced during this project phase?
        i. How were these challenges tackled? Which strategies were used and why?
        ii. Who were the key players in tackling these challenges?
    e. How long (months or years) did it take you to progress to the next project phase (i.e., planning and design)?
    f. Which factors facilitated progress from this phase to the next project phase (i.e., planning and design) and why?
    g. Which factors hindered progress from this phase to the next project phase (i.e., planning and design) and why?
3. Please describe the main activities performed and decisions made during the **planning and design phase**.
    a. What were the main activities performed and major decisions made during this phase?

        i. Who were the key players in making major decisions during this phase?
   b. What were the main challenges faced during this project phase?
        i. How were these challenges tackled? Which strategies were used and why?
        ii. Who were the key players in tackling these challenges?
   c. How long (months or years) did it take you to progress to the next project phase (i.e., construction)?
   d. Which factors facilitated progress from this phase to the next project phase (i.e., construction) and why?
   e. Which factors hindered progress from this phase to the next project phase (i.e., construction) and why?
4. Please describe the main activities performed and decisions made during the **construction phase**, which is the execution phase of the project.
   a. When did construction start (year/month)?
   b. What were the main activities performed and major decisions made during this phase?
        i. Who were the key players in making major decisions during this phase?
   c. What were the main challenges faced?
        i. How were these challenges tackled? Which strategies were used and why?
        ii. Who were the key players in tackling these challenges?
   d. Which factors facilitated progress from this phase to the next project phase (i.e., operation) and why?
   e. Which factors hindered progress from this phase to the next project phase (i.e., operation) and why?
   f. [If operational i.e. successful] When did the project first become operational (year/month)?
   g. [If attempted] At what stage was the project stalled, postponed or abandoned?
        i. What were the last major decisions made before this?
        ii. What were the key barriers?
5. Overall, do you consider the project to be successful? Why or why not?
6. Did the reasons for implementing reuse change over the course of the project?
7. What would you want to change about the current approach to water reuse?
8. What would you keep/not change if you did the project again?
9. Are there any additional comments and/or clarifications you would like to make about the water reuse project or any of the questions in this interview?

Interview 2: Stakeholder Involvement and Engagement

(90 minutes)

I would like to ask some questions that will provide an overview of the project.

1. Please describe your role in the water reuse project at your utility, including your title and main responsibilities (past and present).
2. (if implemented) What were the key actions or decisions that enabled the success of this project?
3. What were the main challenges and lessons learned over the course of this project?
    a. How were the challenges tackled? What strategies were used?

I would now like to talk about stakeholder engagement for the project.

External stakeholder engagement.

4. Which stakeholders were engaged?
    a. For each stakeholder, how were they engaged with the project at each stage and what was the main goal of engaging them?
    b. What type of input/involvement did each type of stakeholder have?
    c. Of the stakeholders engaged, do you feel any particular groups or engagement processes were particularly helpful or hurtful for the project?

Public water consumer perception and acceptance.

5. Does the utility collect data on public perception from the water consumer community?
    a. If yes, can you describe how, when, and what type of data was collected?
    b. How did past experience or other water reuse projects influence public opinion and perception?
    c. How did you gauge public (i.e. water consumer) trust in the utility?
    d. Did you collect or use information on community demographics?
        i. If yes, what information was collected?
        ii. In what ways was this information on water consumer demographics used?

Public education and awareness programs.

For these next questions, I am going to ask about public education and awareness programs and project marketing. First, the education programs.

6. What were the main goals of such a program?
    a. What methods were used to disseminate information to or educate the public?
    b. How did you decide which method(s) to use?

c. Which method(s) was most effective? Why do you think they were more effective?

d. How was the project branded/framed to the public?

i. Which methods were used?

ii. What terminology was used? How was its effectiveness determined?

e. Which method(s) was most effective? Why do you think they were more effective?

f. Out of education programs or project branding, which method was found to be most effective in communicating to the public water consumers about the project?

i. How did you determine this?

Open-ended questions.

7. Do you consider the project to be successful? Why or why not?
8. Looking back, are there any aspects of the approach(es) used to engage with stakeholders that you would change?
    a. If yes, what are these?
9. What would you keep/not change from your approach to stakeholder engagement or education for another potable reuse project?
10. Are there any additional comments and/or clarifications you would like to make about the potable reuse project or any of the questions in this interview?

Interview 3: Potable Reuse Regulations, Laws and Guidelines

(90 minutes)

I would like to ask some questions that will provide an overview of the project.

1. (interviewee role) Please describe your role in the water reuse project at your utility, including your title and main responsibilities (past and present).
2. (if implemented) What were the key actions or decisions that enabled the success of this project?
3. What were the main challenges and lessons learned over the course of this project?
    a. How were the challenges tackled? What strategies were used?

I would now like to talk about relevant regulations, laws and guidelines for the project, including requirements for operator training and workforce development.

4. What were the relevant regulations, laws and/or guidelines?
    a. As you discuss, could you please note the governmental level, such as state or local?
    b. How did they help or hinder the project?
    c. Did the level of regulations or policies (i.e. whether they are at state or local level) affect the ease/difficulty of project implementation? In what ways?
    d. Are there additional regulations that have helped or hindered the project?
    e. What were the permitting requirements for this project?
        i. How easily could you acquire permitting for operation of the plant?
        ii. What steps were involved in this?
        iii. Did you encounter any challenges? Please specify.
        iv. How did you tackle them?
5. If there was one thing you would like to change about the current state of relevant regulations, what would it be?
    a. What are some benefits of the current state of regulations and laws?
6. For operators. What background and experience are required for *operators* working on this project?
7. For utility management. What background and experience are required for *utility management* working on this project?
8. What on-going training or professional development, if any, is required? i.e. how were utility staff trained for water reuse once on-boarded?
9. If there was one thing you would like to change about the current state of approach to staff training at your utility, what would it be?

    a. What are some benefits of the current approach to utility staff training?

10. Do you consider the project to be successful? Why or why not?
11. Are there any additional comments and/or clarifications you would like to make about the potable reuse project or any of the questions in this interview?

Interview 4: Funding and Resources & Development

(60 minutes)

I would like to ask some questions that will provide an overview of the project.

1. (interviewee role) Please describe your role in the water reuse project at your utility, including your title and main responsibilities (past and present).
2. (if implemented) What were the key actions or decisions that enabled the success of this project?
3. What were the main challenges and lessons learned over the course of this project?
    a. How were the challenges tackled? What strategies were used?
4. Funding for capital and O&M and external funding. What are the total and annual capital costs and annual O&M costs?
    a. Is the project able to recover all capital costs?
        i. What were/are the funding sources? (Amounts/percentage)
    b. Is there enough revenue to cover annual O&M costs?
        i. What were/are the funding sources? (Amounts/percentage)
    c. If no, what is/was the funding deficit?
        i. If unknown, would you be able to share your financial records?
    d. What type and amount of public funding, if any, was available from the government?
5. What are the water rates?
    a. How were these set?
    b. Is public willingness-to-pay an important consideration when determining water rates?
    c. Is affordability an important consideration when determining water rates?
    d. Are there any rate-setting policies or guidelines applicable for this project? If yes, which of those were used and in what way?
6. Potable reuse is the most cost-effective alternative.
    a. Was a cost-benefit analysis performed?
        i. If yes, what considerations (e.g. environmental, social, economic impacts) were included?
        ii. If yes, which benefits were major drivers for implementing this water reuse project?
7. If there was one thing you would like to change about the approach to funding for water reuse and management, what would it be?
8. If there was one thing you would like to change about the cost or expense needs for water reuse and management, what would it be?
9. What would you not change if you did the project again?

Existing infrastructure.

10. What is the timeline of the project from proposal/initiation to implementation (for implemented) or current stage (attempted)?
    a. What were the major milestones?
11. How much existing infrastructure was used to implement reuse at your facility?

a. What additional resources were required to implement the project?
b. How easy or challenging was it to acquire these resources and subsequently implement the project?

Open-ended questions.

12. If there was one thing you would like to change about the approach to acquiring resources for water reuse and management, what would it be?
    a. For time?
    b. For ease of implementation?
13. What would you not change if you did the project again?
14. Do you consider the project to be successful? Why or why not?
15. Are there any additional comments and/or clarifications you would like to make about the water reuse project or any of the questions in this interview?

Table S2. Overview of semi-structured interviews conducted by case study, including the number of interviews and interviewee roles (total n = 60).

Case No.	*Total interviews*	*Utility project managers*	*Operations and maintenance staff*	*Engineering consultants*
1	3	2	1	0
2	6	4	2	0
3	4	3	1	0
4	3	2	1	0
5	4	2	2	0
6	6	4	1	1
7	3	2	1	0
8	3	2	1	0
9	5	3	1	1
10	4	2	2	1
11	3	1	1	1
12	4	3	0	1
13	3	1	2	0
14	2	1	0	1
15	2	1	0	1
16	5	2	2	1

S3. Extended fsQCA Analytical Procedure

S3.1. Preliminary Minimization and Removal of Causal Conditions

First, we assembled a list of hypothesized causal conditions from a systematic literature review identifying facilitators and barriers to potable reuse.[2] However, based on case knowledge, we removed conditions based on: (i) lack of variation for a condition across all cases ('domain' or 'remote' conditions in fsQCA);[3,4] or (ii) case knowledge dictated that they weren't major drivers of success or failure.

Several conditions frequently hypothesized as decisive in the success of potable reuse projects and posited as such in existing literature[2,5,6] did not emerge as consistently necessary or sufficient in the final fsQCA even though they were included in the initial fsQCA. They included: drought, discharge compliance, choice of treatment technology, stringency of the permitting regulatory environment, public water consumer decision-making participation, public water consumer demographics or economic feasibility, and relative cost-effectiveness of potable reuse. Their absence does not mean that these conditions were irrelevant but rather that they did not differentiate success in a systematic way across the cases. Therefore, we treated these conditions as domain/remote rather than utility-controllable causal conditions.

For example, while an acute drought crisis was a powerful motivator for Cases 1, 4, 5 and 7, other cases were driven by entirely different pressures including long-term supply reliability. Case 2 was motivated by regional groundwater and surface water discharge compliance legislation to slow land subsidence and fend off saltwater intrusion. Cases 3 and 9 were motivated by state cease-and-desist orders that limited river diversions of wastewater discharge in different states. Case 6 was motivated by the desire to eliminate reliance on imported water enabled by regulatory changes that permitted expanded reuse water use for spreading grounds. Case 8 was catalyzed by

the recent adoption of potable reuse regulations and the availability of sufficient advanced treatment capacity, enabling implementation in a persistently drought-affected region. The fact that success could be achieved under such varied motivations indicated that the *presence* of a strong driver, rather than its specific *nature*, mattered as a means to initiate these projects but not necessarily as a driver of success. Accordingly, we treated these as domain conditions and did not retain them in the analysis as drivers of success.

Similarly, the choice of treatment technology, specifically, the use of membrane (reverse osmosis)-based advanced treatment (i.e., RO-BAT) versus carbon-based advanced treatment (CBAT) was not a defining factor across cases. Cases 3, 4, 6, 7, 8, and 9 chose a UF-RO-UV/AOP membrane train. Cases 1, 2, and 5 chose a carbon-based advanced treatment train to avoid brine production and were likewise successful. Among attempted cases, Cases 10, 11, and 14 chose a UF-RO-UV/AOP membrane train. Cases 12, 13, 15, and 16 chose a carbon-based advanced treatment train. The viability of both technological approaches demonstrated that success was not contingent on a specific set of unit processes but rather on the project's ability to meet performance targets.

Similarly, every successful case either operated or coordinated a pretreatment and source-control program to manage industrial and commercial discharges upstream of advanced treatment for potable reuse, which, among other arrangements, became part of expanded *committed interagency agreements*. In several states, this was not just best practice but a potable reuse permitting condition, and the stringency of requirements varied across states. Therefore, across cases, pretreatment or enhanced source water control programs were either built into interagency agreements or were necessitated by relevant potable reuse regulations. Accordingly, we treated these programs as domain conditions and did not retain them in the analysis as drivers of success.

Additionally, across cases, public decision-making participation occurred primarily through statutory procedures (e.g., city council actions, ballot measures). Because these procedures lay largely outside utility control, we modelled it as a domain causal condition. For example, in Cases 1, 4, and 5, longstanding citizens' advisory committees, chartered by the city council, provided oversight of municipal decisions, including water and wastewater. Other cases were governed by National Environmental Policy Act (NEPA)[7]-type procedures that codified minimum public participation (e.g., public notice, comment periods, and hearings) as prerequisites for permit issuance. Accordingly, we treated public decision-making participation as a domain condition and did not retain it in the analysis as a driver of success.

Similarly, public water consumer demographics and attitudes (e.g., median household income, average education level, political leaning at the time of the potable reuse project's implementation) were examined in preliminary analyses but showed substantial heterogeneity and no consistent association with success (i.e., they did not differentiate between successful and attempted cases in a meaningful way). Accordingly, we treated them as domain conditions and did not retain them in the analysis.

Economic feasibility has also been consistently cited as a pivotal factor in potable reuse project planning. Accordingly, in many of the above cases, a reuse project moved forward because its projected normalizedcost of water – the total CAPEX per unit volume of water produced – was competitive with or decisively lower than the cost of alternatives. For example, Case 5's \$2.43M/MGD reuse supply equated to only about 1/4 the unit cost of a proposed surface water reservoir, making the choice to implement potable reuse straightforward. Similarly, Case 9's recycled water was delivered to the utility at around \$1,720 per acre-foot, which undercut the cost of a proposed seawater desalination project (estimated \$6,000–\$7,000+ per acre-foot) so much that

the expensive desalination proposal faced opposition for being economically unfeasible, making potable reuse the most competitive option compared on life cycle cost. These cases illustrated how a favorable unitized cost of water drove public and political support: a project was easier to champion when it provided a needed alternate supply at the most reasonable cost. Even in Case 1, where the reuse project produced water at an order of magnitude higher cost than the city's traditional supplies, severe drought risk and limited new water rights made reuse the most expeditious and meaningful solution despite a higher infrastructure integration burden. The utility in Case 1 mitigated cost concerns by scaling the project for future demand and partnering with regional agencies to share costs and commit to regulatory agreements, thus providing evidence of how *committed interagency agreements* can alleviate cost differentials across projects, still making them the most cost-effective alternative. In general, communities with urgent water needs were likely to proceed with potable reuse even at higher unit costs, because the cost of *not* having water, importing water or alternatives such as desalination, outweighed financial costs. Accordingly, we *treated economic feasibility as* a domain condition and did not retain it in the analysis.

Finally, while all projects operated in complex regulatory environments with unique requirements (e.g., piloting), some cases were successful with specific state-level potable reuse regulations in place while others were approved on a case-by-case basis, and the degree of regulatory stringency itself was not a driver of success. For example, Cases 1, 2, 4, and 7 navigated successful implementation with no specific state potable reuse regulations at the time, which yielded project planning and target performance flexibility, while others like Cases 3, 5, 6, 8, and 9 succeeded under a pre-established high-stringency regulatory regime with a highly prescriptive framework. For example, we excluded piloting from the final fsQCA causal model because its implementation was largely regulation- dependent and history-dependent rather than strategy-

dependent. Across cases, pilots ranged from no formal pilot (Cases 1, 6, and 8); tobrief verification pilot testing (Case 4, 5, and 7); to multi-year pilot demonstration facilities (Cases 2, 3, and 9). Some pilots were required pre-decision (Cases 2, 3, 4, 5, 7, and 9), while other pilots occurred after commissioning, to validate performance (Cases 1, 6, and 8). Because this variation reflected regulatory design and legacy plant history more than utility-level discretion, we treated piloting as a domain/regulatory condition rather than a utility-level controllable causal condition.

Similarly, operator training and certification requirements varied widely across cases—in credential type, grade, and reuse-specific endorsements—and were set primarily by state programs and project permits rather than utility discretion. Given this regulatory heterogeneity and limited controllability, we treated operator training and certification regulatory requirements as a contextual compliance condition rather than a causal condition. Accordingly, we determined that while regulatory variation across states could affect project implementation ease, it could not be consistently calibrated as a causal condition since every successful cases ultimately obtained all necessary permits. The key determinant of success therefore was not the existence of regulations or the extent of regulatory complexity/stringency but the utility's capacity to navigate the existing regulatory framework, a capacity enabled by the core conditions and strategic pathways identified in this analysis. Accordingly, we treated *regulatory stringency* as a domain condition and did not retain it in the analysis as a driver of success.

Overall, our analysis thus suggests that potable reuse success depends less on exogenous pressures and more on utilities' capacity to institutionalize core management practices and align project strategy with available organizational, financial and social resources.

S3.2. Detailed Definitions and Calibrations of Causal Conditions

Definitions, scoring rationale and calibrations for each causal conditions are provided below, in the order listed in Table 1.

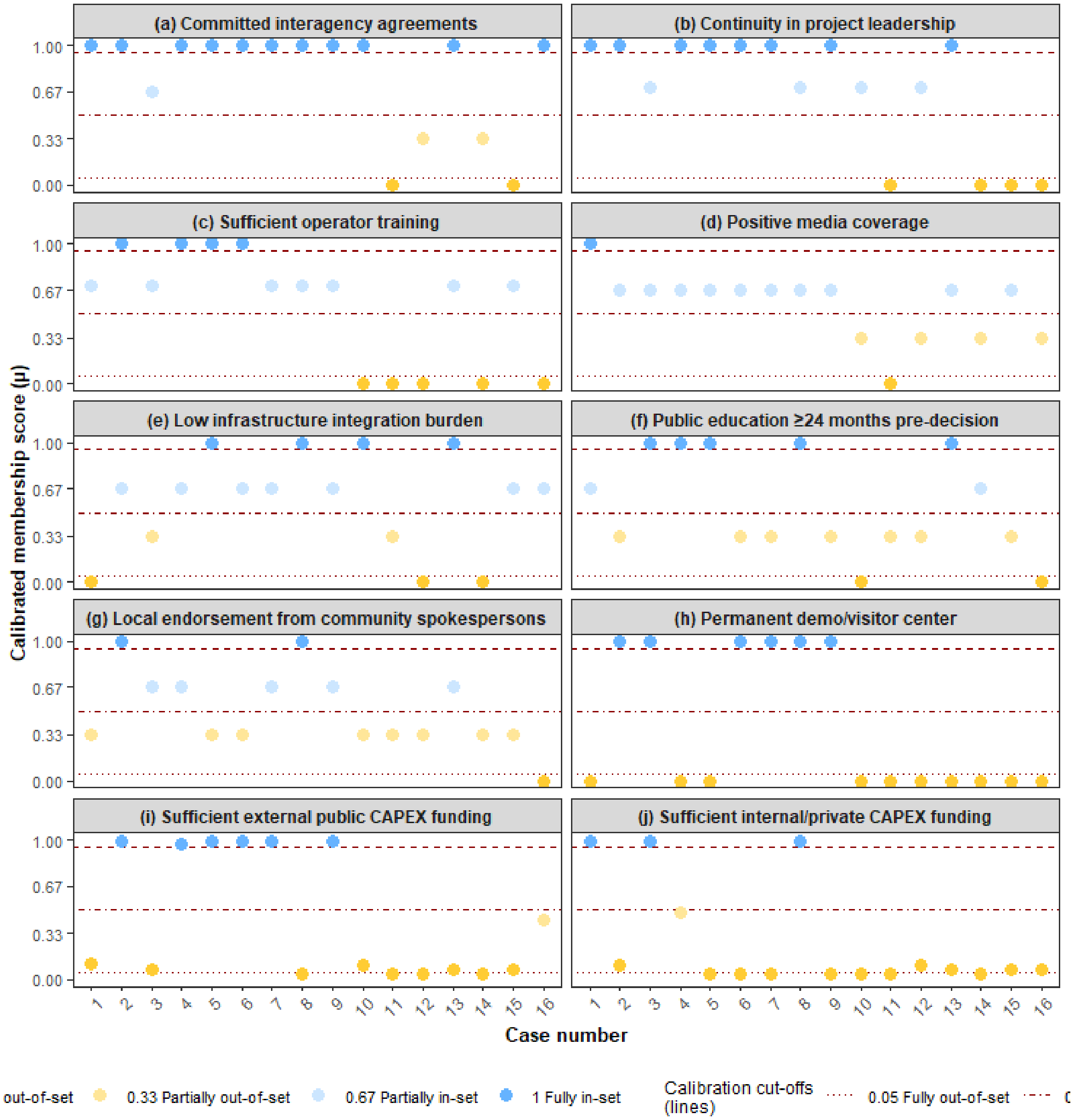

Figure S1. Distribution of calibrated set membership scores for each causal condition and all 16 cases.

<u>Committed Interagency Agreements</u>

This factor is defined as executed, binding agreements among the key agencies (i.e., the utility implementing potable reuse and neighboring retailers/wholesalers, beneficiary customers

like golf courses/industry) that assign roles, responsibilities, and resource commitments for the potable reuse project. Case knowledge dictates that interagency agreements can help projects spread water consumer rate impact and political risk across multiple beneficiaries of potable reuse water, increase board and regulator acceptability, and align incentives across agencies that control key infrastructure needed to implement potable reuse projects successfully. Conversely, when these agreements are needed but missing (e.g., in some attempted cases), projects remain vulnerable to vetoes from regional beneficiaries even if external grants or loans exist. Committed interagency agreements are not needed when a single legal entity (e.g., a city utility) controls the wastewater source, the advanced potable reuse treatment facility, the product-water tie-in. and the recharge/brine route, and there is no distinct beneficiary group expected to contractually co-fund the project. Committed interagency agreements are needed when any independent partner controls a critical asset (e.g., a separate drinking-water retailer, a groundwater/spreading manager, or a conveyance owner), or when beneficiaries (e.g., golf courses, industry, wholesale customers) are expected to underwrite a material portion of project CAPEX.

Scoring rationale:

In-set membership is when cases had executed, binding agreements they needed through established processes that allocated financing and jurisdictional roles and responsibilities across all independent partners controlling critical assets. Cases where cost-sharing was structurally unnecessary (e.g., a single legal entity implementing potable reuse) are also fully in this set because the barrier that these agreements resolved did not exist for these cases. Mostly in-set membership was when executed agreements covered most but not all required partners/assets, with the remaining agreements posing low project veto risk. Mostly out-of-set of membership was when negotiations were underway (e.g., letters of intent), but material terms were unsettled (e.g., delivery volumes, schedule alignment), or agreements existed but were conditional and did not yet secure access to a critical asset or a material beneficiary contribution. Out-of-set membership was when committed interagency agreements were needed but missing at the decision point and there were no concrete, enforceable resource commitments.

Table S3. Indirect Calibration for *Committed Interagency Agreements*.

Fuzzy Score (μ)	Description

1.00	The implementing utility needed committed interagency agreements, and had executed, board-approved, legally enforceable agreements with all critical agencies (e.g., wastewater producer/receiver, groundwater or surface water authority, conveyance asset owners, wholesale suppliers, regulatory co-permittees), or when such agreements were not needed (e.g., for projects wholly within a single jurisdiction).
0.67	The implementing utility needed committed interagency agreements and had most binding agreements executed but one or two binding elements (e.g., cost-share specifics, conveyance easements) remained conditional or short-term.
0.33	The implementing utility needed committed interagency agreements, but the record showed only draft memorandums-of-understanding (MOUs), term sheets, or conditional approvals and no long-term agreements had been committed.
0.00	The implementing utility needed committed interagency agreements, but no binding agreements existed beyond non-binding letters of intent or draft MOUs, key elements remained unresolved (e.g., volumes, costs, rights-of-way, permit responsibilities), or material contestation (e.g., active disputes or pending litigation) undermined project execution.

<u>Continuity in Project Leadership</u>

This factor is defined as the presence of stable, visible management champions (internal to the implementing utility) with the authority and technical literacy to steer potable reuse projects from initiation through design, permitting, financing, construction, and commissioning. In practice, this means one clearly accountable lead or a small core team of decision-making engineers that stayed in post across project phases, coordinated decisions, and maintained alignment among internal divisions and external partners. For potable reuse, where projects span years, require complex treatment integration, multi-agency coordination, and sustained public and regulatory engagement, across cases, project leadership continuity was repeatedly linked to faster decision cycles, fewer redesigns, better risk management, and durable stakeholder trust. Conversely, leadership turnover was discussed as a barrier to implementing by eroding institutional memory, producing decision churn (e.g., repeated revisiting of scope, permitting strategy, funding plan), slowing regulatory approvals, disrupting outreach narratives, and heightening project veto risk from boards or partners.

<u>Scoring rationale:</u>

In-set membership is when the utility project leadership remained consistent throughout the project timeline, either via a dedicated project lead or a core team of decision-making

engineers. Out-of-set membership is when there was high attrition in utility project leadership overseeing the project. Intermediate scores reflect the different levels of continuity and attrition in utility project leadership.

Table S4. Indirect Calibration for *Continuity in Project Leadership*.

Fuzzy Score (μ)	Description
1.00	Project leadership remained substantively continuous from initiation through construction/commissioning, either a single accountable project lead or a small core team of decision-making engineers who retained formal decision rights across phases, with documented handoffs (if any) that preserved authority and institutional memory (e.g., succession plan/phase-gate charter) and no material decision churn.
0.7	Project leadership experienced some attrition during the project's timeline, e.g., one leadership change occurred but with an orderly transition and limited rework, or the core team remained intact while a secondary lead rotated without affecting project scope, permitting strategy, or financing.
0.00	Project leadership did not remain continuous during the project's timeline. An advocate emerged during the initiation phase but left (e.g., retirement) before key project milestones were achieved, leading to changed utility priorities, resulting in persistent decision churn, stalled project approvals, or major project scope rework.

<u>Sufficient Operator Training</u>

This factor encompasses the degree to which operations staff (e.g., certified water/wastewater treatment operators, maintenance technicians, and instrumentation/controls technicians, often with overlapping roles) possessed, or could rapidly obtain, the certifications, competencies, and hands-on experience required to run advanced potable reuse safely and reliably, and operators were embedded into the project early enough to develop ownership of the process. Across the literature and our cases, there was emphasis on early operator involvement in design reviews, piloting, and commissioning that helped build procedural fluency, hazard awareness and response, and cultivate a safety culture that reduced human-error incidents; it also created trusted internal champions who could explain treatment barriers to boards, regulators, and the public with credibility. Utilities that formalized training plans pre-startup, cross-trained water and wastewater operators, and tied process refreshers to permit/log-removal requirements reported smoother transitions to swift troubleshooting and fewer alarm cascades which are outcomes that, in turn, strengthened external trust through consistent compliance and transparent operator narratives. By

contrast, in some cases, ad-hoc or late operator training left potable reuse plants dependent on consultants and undermined both operator and stakeholder confidence in the system.

Scoring rationale:

In-set membership is when a structured operator training program was in place before startup/commissioning, operators held required certification grades for the facility class, or operators were cross-trained on critical treatment and controls via hands-on training. Out-of-set membership was when the operations staff were not certified and trained in operating and maintaining a potable reuse plant and no such training was provided. Intermediate scores reflect an absence of certified/trained operations staff, but when training programs were administered to help with operator workforce development.

Table S5. Indirect Calibration for *Sufficient Operator Training*.

Fuzzy Score (μ)	Description
1.00	Fully trained and experienced operations staff. At least 1 lead operator already held advanced treatment certification. Operations staff had significant experience (>5 years) working with advanced water treatment systems such as through hands-on pilot exposure. Comprehensive training programs were available for all other operations staff such that they were able to acquire advanced certifications with support from the utility.
0.7	Moderately trained operations staff. The operations staff were not certified to operate and maintain a potable reuse plant but comprehensive training programs were available such that the staff were able to acquire advanced certifications within the project timeline. Operators had minimal to moderate experience (2-5 years) working with advanced water treatment systems such as through hands-on pilot exposure.
0.00	Minimally trained or untrained operations staff. Basic training was provided but was limited to meeting minimum regulatory requirements addressing immediate compliance needs. Operators relied on basic operational knowledge without specialized or advanced certifications or training, and there was little to no hands-on pilot exposure. Operators had minimal (<2 years) to no experience working with advanced water treatment systems.

Positive Media Coverage

This factor is defined as the extent to which local mainstream media (e.g., print/online outlets, TV/radio) framed potable reuse in supportive or neutral terms (e.g., water resiliency) versus oppositional frames (e.g., “toilet-to-tap”). Existing research shows that media act as

agenda-setters and frame interpreters that can normalize potable reuse, amplify trusted messengers, and build confidence. Conversely, negative media frames can entrench public opposition and heighten project veto risk. Case knowledge also consistently linked constructive or neutral media coverage to higher acceptance and smoother decision cycles, while sensational or conflict-focused narratives correlated with project delays or cancellation. Utilities emphasized the value of media monitoring and proactive messaging to counter misperceptions, secure public acceptance and sustain public trust.

Scoring rationale:

In-set membership is when across the project's critical decision window (e.g., initiation→authorization), the dominant local media tone was supportive or neutral, with accurate technical framing (e.g., multiple barriers, monitoring, peer projects) and limited recurrence of stigmatizing framing; and did not produce sustained controversy or measurable delays. Out-of-set membership was when oppositional framing dominated (persistent "toilet-to-tap" headlines, conflict narratives), eroding trust and contributing to stalls, failed votes, or withdrawals. Intermediate scores encompass media coverage with mixed, leaning supportive and mixed, frequently negative framing.

Table S6. Indirect Calibration for *Positive Media Coverage.*

Fuzzy Score (μ)	Description
1.0	Local media coverage was predominantly supportive or neutral, with minimal use of negative frames such as "toilet-to-tap."
0.67	Local media coverage was largely neutral/constructive but included isolated negative pieces or short-lived "toilet-to-tap" references that were effectively counter-framed (e.g., op-eds, expert interviews) without observable schedule impacts.
0.33	Local media coverage was largely negative and oppositional frames dominated across outlets or over time, prompting additional outreach, board hesitancy, or minor re-scoping, even if the project ultimately advanced.
0.0	Local media coverage was predominantly negative, with sustained pejorative frames or repeated stories about project opposition eroded public trust and contributed to project stalls, failed votes, or withdrawals.

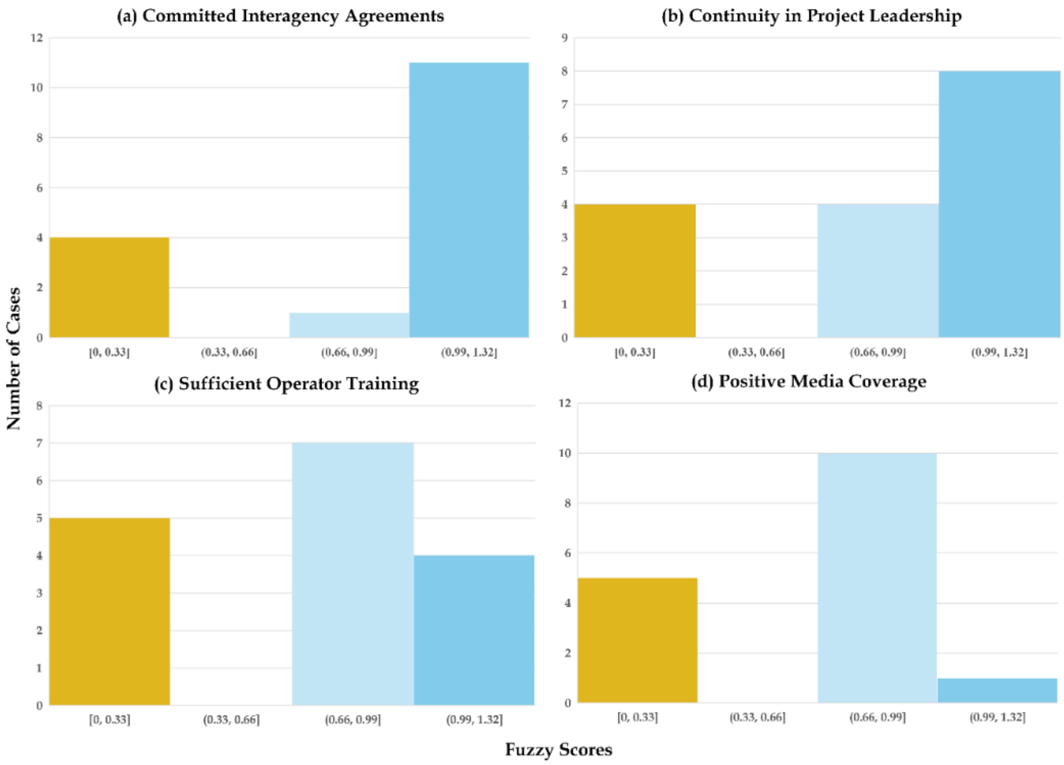

Figure S2. Distribution of calibrated membership scores for the four conditions common across all success pathways (P1-P4): (a) Committed Interagency Agreements; (b) Continuity in Project Leadership; (c) Sufficient Operator Training; (d) Positive Media Coverage.

<u>Low Infrastructure Integration Burden</u>

This factor is defined as the amount of new conveyance and sitework infrastructure, such as pipes, pumps, and storage, that needed to be built to link the wastewater source to the potable reuse facility and then deliver the potable reuse water to its destination, which could be an environmental buffer (for indirect potable reuse) or the drinking water supply itself (for direct potable reuse). A higher infrastructure integration burden typically implies greater capital cost (e.g., by requiring more piping and land acquisition), logistical complexity (e.g., longer routes require more extensive planning, engineering, and construction management), and a longer project timeline (e.g., securing the legal rights to build across miles of public and private land can be a major source of delays in large infrastructure projects). Conversely, co-located systems and short tie-ins reduce cost and delay risks.

<u>Scoring rationale:</u>

In-set membership is for cases with very short or co-located systems that typically needed fewer easements and permits, stayed within one jurisdiction, and added minimal pumping. Out-of-set membership is for cases with extremely high-burden integration of conveyance systems (e.g., long multi-jurisdictional pipelines with major crossings and new lift/storage assets). Intermediate scores reflect moderate and minimal amount of new conveyance needed.

Table S7. Indirect Calibration for *Low Infrastructure Integration Burden.*

Fuzzy Score (μ)	Description
1.0	The potable reuse system was co-located or adjacent to existing facilities such that no off-site conveyance was needed, or the combined new wastewater-drinking water alignment was 2 miles or less (e.g., the potable reuse system was on the same or neighboring site as the wastewater source and either the potable reuse water tie-in (DPR) or the environmental buffer and potable reuse water intake (for IPR)). The integration burden was minimal.
0.67	The project required a modest amount of new conveyance—2-15 mi—typically within the implementing utility’s service area and mostly along existing transportation or utility corridors. The integration burden was noticeable but not dominant in cost or schedule risk.
0.33	The project had to build between 15-30 mi of new conveyance, often across multiple neighborhoods or jurisdictions and with several pump stations and major crossings. Right-of-way acquisition and environmental permitting became significant drivers of cost, timeline, and stakeholder complexity. The integration burden was high enough to influence project feasibility and phasing.
0.0	The project depended on more than 30 miles of new conveyance, creating a regional-scale network with numerous crossings, and substantial capital investment. The integration burden was high enough to require major funding and coordination to proceed.

<u>Public Education ≥ 24 Months Pre-decision</u>

This factor is defined as a sustained public education and awareness program that began at least 24 months before the pivotal project authorization decision (e.g., council/board approval, bond/loan award) and ran continuously through that decision. Drawing on potable reuse communications literature and our case knowledge, early education lead time enabled public familiarity and risk literacy to develop gradually, normalized potable reuse, allowed repetition and message testing, built trusted messengers, and reduced the perception of reactive, late-stage communications or post-decision justifications. Effective programs paired clear safety evidence (multi-barrier treatment, log-removal credits, monitoring/response plans, source control) with tangible experiences (pilot tours, operators as community liaisons). Conversely, compressed or

post-decision public education timelines were linked to public suspicion, reactive communications, organized opposition, and decision delays. In practice, qualifying programs showed a documented plan and budget, a steady cadence, tailored materials for distinct audiences (e.g., residents, businesses, civic groups), and evidence of learning loops (e.g., tracking questions, refining messages). This factor specifically captured the timing and sustained nature of education rather than any single channel.

<u>Scoring rationale:</u>

In-set membership encompasses cases that implemented public education and awareness programs at least 24 months before the project authorization decision and maintained a steady cadence of public engagement, reflecting guidance to embed communication well before process selection and permits so public could process risk information early. Out-of-set membership is for cases that implemented public education and awareness programs post-decision or held brief information sessions. Intermediate membership is when public education and awareness programs were implemented in the 1-23 months before the project authorization decision capturing substantial but less institutionalized programs.

Table S8. Indirect Calibration for *Public Education ≥ 24 Months Pre-decision.*

Fuzzy Score (μ)	Description
1	Public education began ≥24 months before the first project authorization decision, culminating in a well-informed decision process without major last-minute communication crises.
0.67	Public education began 12-23 months before the first project authorization decision with no material decision delays attributable to communication.

0.33	Public education began 1-11 months before the first project authorization decision and decision makers faced noticeable pushback or extended hearings linked to information deficits even if some corrective actions were taken.
0	Public education was primarily post-decision (initiated after a major vote/permit filing), was largely reactive (e.g., after opposition emerged), and contributed to project delays or cancellation.

Local Endorsement from Community Spokespersons

This factor encompasses when a project secured public acceptance by using endorsement from community spokespersons such as via local business partnerships, environmental conservancy groups, and academic and public health organizations to translate utility water quality claims into relatable experiences. These endorsements co-created public outreach events (e.g., purified water tastings, beer showcases, school demos) and publicly vouched for the safety and community value of potable reuse water. The literature shows that public acceptance of potable reuse hinges not only on data but on source credibility and messenger trust; pairing third-party validation with familiar, high-trust local voices, experiential events (e.g., tastings/tours), and consistent, transparent messaging increases willingness to try and support potable reuse, reduces "yuck factor," and builds durable social legitimacy. Case knowledge echoed this finding: utilities leveraging expert panels plus local anchors (e.g., Pure Water Brew initiatives) reported fewer public perception challenges and steadier board and regulatory support than those relying on technical reports alone.

Scoring rationale:

In-set membership is when the project had active and explicit endorsements from trusted local spokespersons endorsing potable reuse safety and community value. Out-of-set membership is when the project did not have endorsements from trust local spokespersons. Intermediate scores reflect varying levels of community spokespersons' endorsements.

Table S9. Indirect Calibration for *Local Endorsement from Community Spokespersons*.

Fuzzy Score (μ)	Description
1.0	The project had endorsements from trusted local spokespersons (e.g., community businesses, civic/environmental groups, schools/health agencies) who actively co-created public outreach by running visible,

	recurring, and co-branded activities (e.g., tastings, brewery/coffee showcases, school demos, tours) with explicit public endorsements of potable reuse safety and community value, documented in earned media, partner statements, and event materials, and reaching multiple audiences over time.
0.67	The project had endorsements from trusted local spokespersons but these were narrower in scope (e.g., one or two community partners, or a limited run of co-created events that nevertheless produced clear public-facing endorsements).
0.33	The project had only ad-hoc or one-off endorsements from trusted local spokespersons (e.g., a single event, passive venue hosting, or social posts without explicit endorsement), reaching a small fraction of the community with minimal influence on public acceptance.
0.0	The project had no endorsements from trusted local spokespersons and project messaging was utility-level only.

Permanent Demo/Visitor Center

This factor is defined as whether the utility created a dedicated, permanent public-facing facility (i.e., a visitor or demonstration center) with recurring public tours, exhibitions, and education, beyond occasional plant tours. Across both the literature and our cases, permanent demo/visitor centers are frequently cited as a visible transparency commitment that (i) makes treatment barriers tangible, (ii) normalizes potable reuse through repeated, trusted contact, (iii) equips staff and community partners with a consistent curriculum, and (iv) sustains earned trust with boards, regulators, media, and residents over multi-year timelines. At the same time, case evidence shows demo/visitor centers are not necessary in all successes; other pathways (e.g., credible local endorsements or early, structured education programs) can also secure public acceptance. Here, this factor captures the presence of a durable, institutionalized outreach asset, not overall outreach intensity.

Scoring rationale:

In-set membership is when a permanent, branded facility existed with a regular public tour schedule, purpose-built exhibits explaining the potable reuse process, and consistent staffing delivered a repeatable curriculum. Out-of-set membership is when no dedicated public visitor center existed.

Table S10. Indirect Calibration for *Permanent Demo/Visitor Center*.

Fuzzy Score (μ)	Description
1	The utility operated a permanent demo/visitor center with a standing schedule of public tours or open hours, branded exhibits, and repeat programming tied to the potable reuse project.
0	The utility did not operate a permanent visitor/demo center; at most, there were tour opportunities at existing plants or occasional events without a dedicated, public-facing facility.

<u>Sufficient External Public CAPEX Funding</u>

External public CAPEX funding is defined as share of total capital expenditures for the potable reuse project that is provided by public entities other than the implementing utility via competitive or administratively awarded grants and/or subsidized public loans. Examples in the U.S. include U.S. Bureau of Reclamation Title XVI grants, state or federal SRF/WIFIA loans (highly subsidized rates), state revolving grants; and local or regional agency capital contributions. This type of funding typically covers planning, design, construction, commissioning, and major rehabilitation when budgeted as CAPEX, and excludes routine O&M costs; rate-funded operating budgets; short-term pilots not capitalized; purely private equity; utility-issued revenue bonds controlled by the implementing utility (these count as *internal/private*—see below). Higher *external public CAPEX funding* reflects dependence on external public capital cycles and requirements (e.g., cost-effectiveness demonstrations, lengthy approvals). It is decision-relevant because it can enable projects with constrained internal monetary resources yet can slow projects when awards are uncertain or delayed.

<u>Scoring rationale:</u>

In-set membership is when *external public funding* covered ≥ two-thirds of CAPEX. Out-of-set membership is when *external public funding* covered ≤5% of CAPEX. Intermediate membership is when *external public funding* covered ≈ one-third of CAPEX.

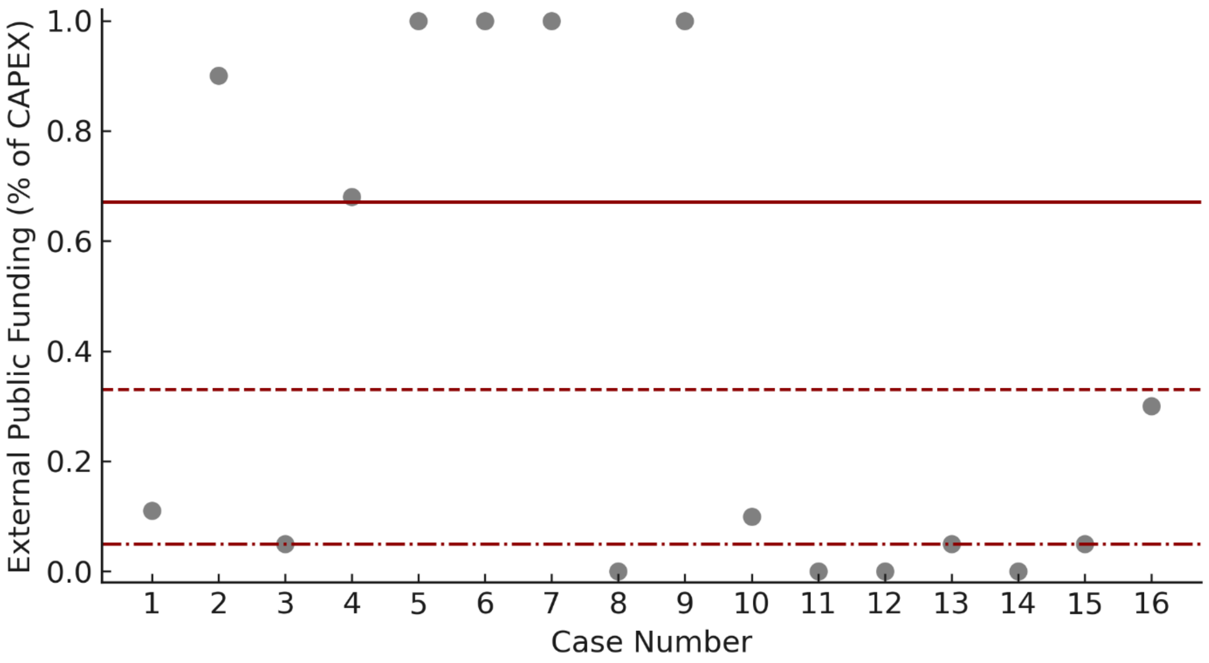

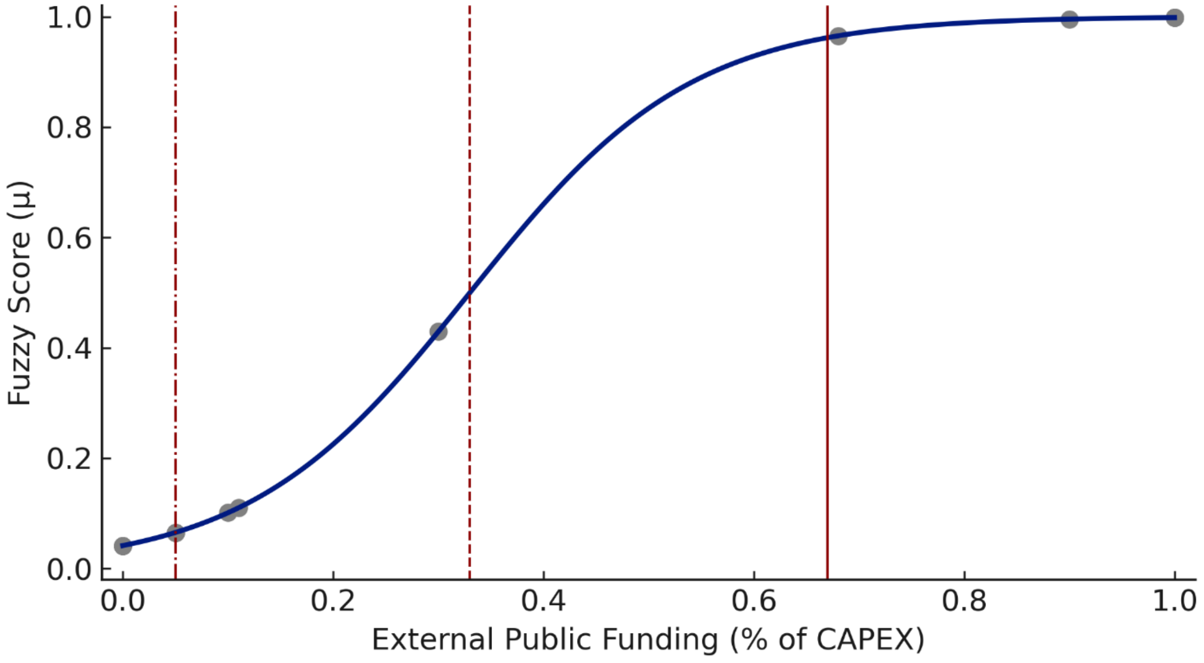

Figure S3. Direct Calibration for *Sufficient External Public CAPEX Funding*. (a) Anchor and crossover points for direct calibration for *Sufficient External Public CAPEX Funding*. The solid line represents the threshold for in-set membership; the dashed line represents the cross-over point; the dashed-and-dotted line represents the threshold for out-of-set membership. (b) Direct calibration for *Sufficient External Public CAPEX Funding*.

Sufficient Internal/Private CAPEX Funding

Internal or private CAPEX funding is defined as share of total capital expenditures for the potable reuse project that is under the implementing utility's or private partner's control, such that the project can proceed without waiting for competitive public awards. It encompasses utility cash reserves, rate-backed revenue bonds, developer/private capital, dedicated system-development charges, and council-approved general obligation bonds. It excludes external public grants and subsidized public loans administered by other public agencies. *Internal/private CAPEX funding* measures whether any capital under utility/private control is ample enough to maintain schedule certainty (planning → design → construction) independent of competitive external awards.

Scoring rationale:

In-set membership is when *internal/private funding* covered ≥ two-thirds of CAPEX. Out-of-set membership is when *internal/private funding* covered ≤5% of CAPEX. Intermediate membership is when *internal/private funding* covered ≈ one-third of CAPEX.

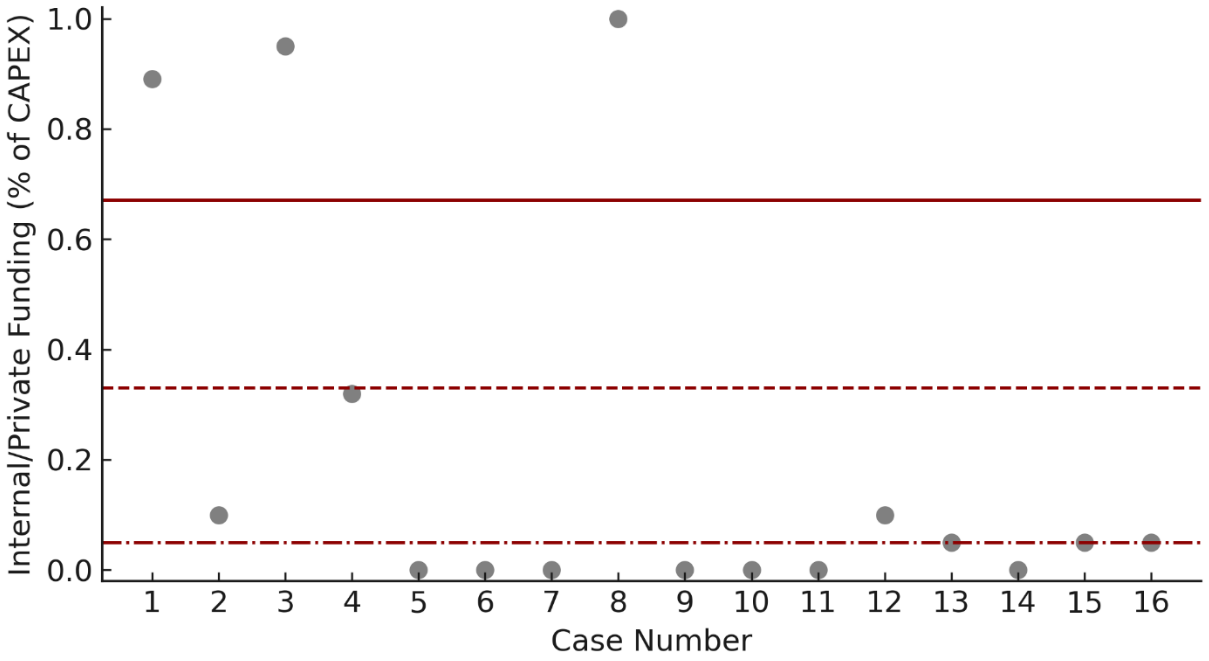

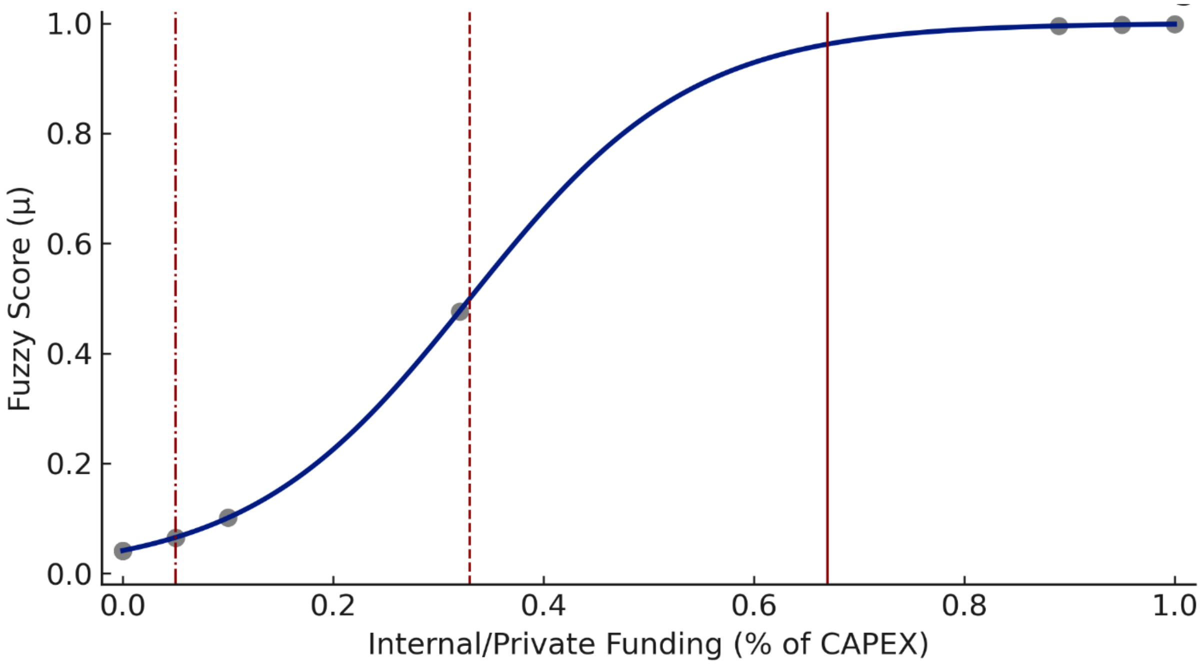

Figure S4. Direct Calibration for *Sufficient Internal/Private CAPEX Funding*. (a) Anchor and crossover points for direct calibration for *Sufficient Internal/Private CAPEX Funding*. The solid line represents the threshold for in-set membership; the dashed line represents the cross-over point; the dashed-and-dotted line represents the threshold for out-of-set membership. (b) Direct calibration for *Sufficient Internal/Private CAPEX Funding*.

S4.3. Step-by-step QCA procedure using fs/QCA 4.1 software

S4.3.1. Process

Software. fs/QCA v4.1 (Windows).[8] All analyses were performed using the graphical user interface (GUI) unless noted.

Data. Rows = cases; columns = calibrated fuzzy-set memberships (0–1) for the outcome **Y** and causal conditions $\mathbf{X_1 \ldots X_k}$.

Step 1. Project setup

i. Open fs/QCA 4.1 → File → Import Data → select .csv, which is the data matrix (Table 2).

ii. Verify causal condition and outcome columns (i.e., all membership scores μ∈[0,1]).

File Variables Cases Analyze Graphs

Case	Succ	Fail	IntFuzz	ExtFuzz	IntegBur	OpTrain	Mgmt	IntAgen	PubEdTim	PubEdVis	Endor	MedCo
1	1	0	0.9951	0.1101	0	0.7	1	1	0.67	0	0.33	
2	1	0	0.1011	0.9956	0.67	1	1	1	0.33	1	1	
3	1	0	0.9972	0.0654	0.33	0.7	0.7	0.67	1	1	0.67	
4	1	0	0.4763	0.9979	0.67	1	1	1	1	0	0.67	
5	1	0	0.0417	0.9983	1	1	1	1	1	0	0.33	
6	1	0	0.0417	0.9983	0.67	1	1	1	0.33	1	0.33	
7	1	0	0.0417	0.9983	0.67	0.7	1	1	0.33	0	0.67	
8	1	0	0.9983	0.0417	1	0.7	0.7	1	1	1	1	
9	1	0	0.0417	0.9983	0.67	0.7	1	1	0.33	1	0.67	
10	0	1	0.0417	0.1011	1	0	0.7	1	0	0	0.33	
11	0	1	0.0417	0.0417	0.33	0	0	0	0.33	0	0.33	
12	0	1	0.1011	0.0417	0	0	0.7	1	0.33	0	0.33	
13	0	1	0.0654	0.0654	1	0.7	1	1	1	0	0.67	
14	0	1	0.0417	0.0417	0	0	0	0.33	0.67	0	0.33	
15	0	1	0.0654	0.0654	0.67	0.7	0	0	0.33	0	0.33	
16	0	1	0.0654	0.4292	0.67	0	0	1	0	0	0	

Step 2. Inspect descriptive set information

iii. Analyze → Statistics → Descriptives to confirm min/max/mean of each set ∈[0,1].

Variable	Mean	Std. Dev.	Minimum	Maximum	N Cases	Missing
IntFuzz	0.259825	0.3685612	0.0417	0.9983	16	0
ExtFuzz	0.4368812	0.4434196	0.0417	0.9983	16	0
IntegBur	0.584375	0.3442559	0	1	16	0
OpTrain	0.55625	0.3936508	0	1	16	0
Mgmt	0.675	0.4085034	0	1	16	0
IntAgen	0.8125	0.3531377	0	1	16	0
PubEdTim	0.540625	0.3521092	0	1	16	0
PubEdVis	0.3125	0.4635124	0	1	16	0
Endor	0.499375	0.2652468	0	1	16	0
MedCov	0.60625	0.2127168	0	1	16	0

Table S11. Table with causal condition name and shorthand used for algorithmic analysis.

Causal Condition	Causal Condition Shorthand Name
Sufficient Internal/Private CAPEX Funding	IntFuzz

Sufficient External Public CAPEX Funding	ExtFuzz
Low Infrastructure Integration Burden	IntegBur
Sufficient Operator Training	OpTrain
Continuity in Project Leadership	Mgmt
Committed Interagency Agreements	IntAgen
Public Education ≥ 24 Months Pre-decision	PubEdTim
Permanent Demo/Visitor Center	PubEdVis
Local Endorsement from Community Spokespersons	Endor
Positive Media Coverage	MedCov

<u>Step 3. Necessity analysis of individual causal conditions</u>

iv. Analyze → Necessary Conditions:

- Select Y as outcome (Succ) and check each X_i and its negation $\sim X_i$.

```
Analysis of Necessary Conditions

Outcome variable: Succ

Conditions tested:
            Consistency    Coverage
IntFuzz     0.414978       0.898393
~IntFuzz    0.585022       0.444591
ExtFuzz     0.689322       0.887527
~ExtFuzz    0.310678       0.310336
IntegBur    0.631111       0.607487
~IntegBur   0.368889       0.499248
OpTrain     0.833333       0.842697
~OpTrain    0.166667       0.211268
Mgmt        0.933333       0.777778
~Mgmt       0.066667       0.115385
IntAgen     0.963333       0.666923
~IntAgen    0.036667       0.110000
PubEdTim    0.665556       0.692486
~PubEdTim   0.334444       0.409524
PubEdVis    0.555556       1.000000
~PubEdVis   0.444444       0.363636
Endor       0.630000       0.709637
~Endor      0.370000       0.415730
MedCov      0.706667       0.655670
~MedCov     0.293333       0.419048
```

Table S12. Analysis of necessity consistency and necessity coverage for each causal condition for the outcome of success, tested for both the presence and absence of the causal condition, arranged in order of decreasing necessity consistency.

**Causal Condition Name**	**Consistency**	**Coverage**
Committed Interagency Agreements	0.96	0.67
Continuity in Project Leadership	0.93	0.78
Sufficient Operator Training	0.83	0.84
Positive Media Coverage	0.71	0.66
Sufficient External Public CAPEX Funding	0.69	0.89
Public Education Months ≥ 24 Months Pre-decision	0.67	0.69

Low Infrastructure Integration Burden	0.63	0.61
Local Endorsement from Community Spokespersons	0.63	0.71
(lack of) Sufficient Internal/Private CAPEX Funding	0.59	0.44
Permanent Demo/Visitor Center	0.56	1.00
(lack of) Permanent Demo/Visitor Center	0.44	0.36
Sufficient Internal/Private CAPEX Funding	0.41	0.90
(lack of) Local Endorsement from Community Spokespersons	0.37	0.42
(lack of) Low Infrastructure Integration Burden	0.37	0.50
(lack of) Public Education Months Pre-decision	0.33	0.41
(lack of) Sufficient External Public CAPEX Funding	0.31	0.31
(lack of) Positive Media Coverage	0.29	0.42
(lack of) Sufficient Operator Training	0.17	0.21
(lack of) Continuity in Project Leadership	0.07	0.12
(lack of) Committed Interagency Agreements	0.04	0.11

- Record necessity consistency and necessity coverage.
  Decision rule: A condition is "(almost) necessary" if consistency ≥ 0.90. We did not drop conditions solely for being necessary; we used this test to aid interpretation.

<u>Step 4. Build the truth table</u>

v. Analyze → Truth Table Algorithm:

- Outcome: Y (success).
- Conditions: select the model set.

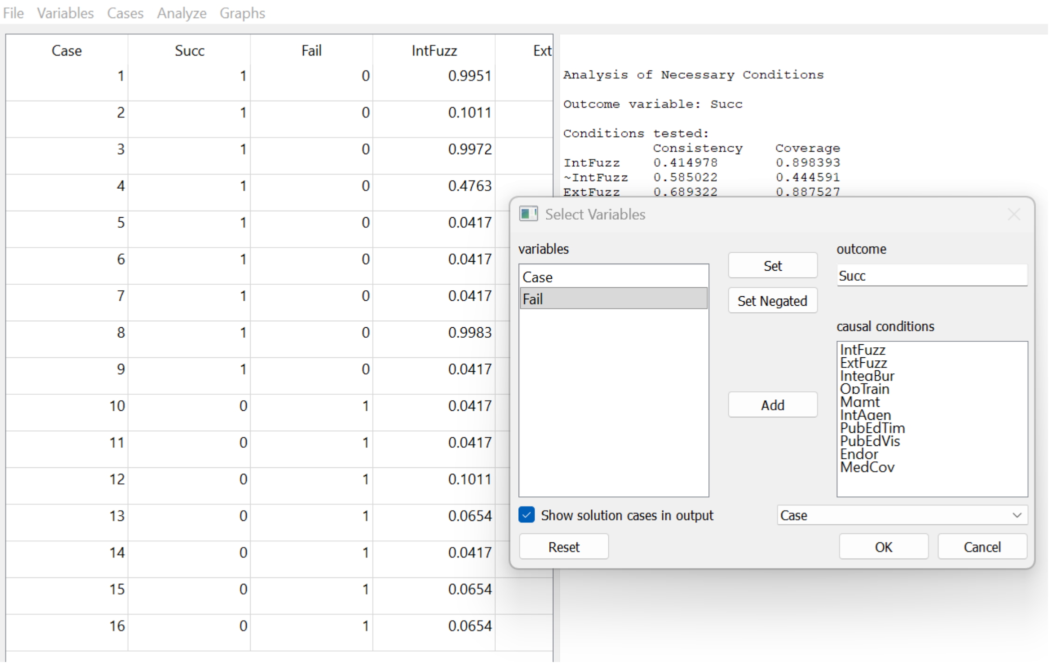

- In the Truth Table window, Edit → Delete and code…
  - Frequency cutoff: set to 1 (each configuration must have ≥1 case).
  - Consistency cutoff: set to 0.80 (baseline); we report sensitivity at 0.75 and 0.85 (Step 7; see below).

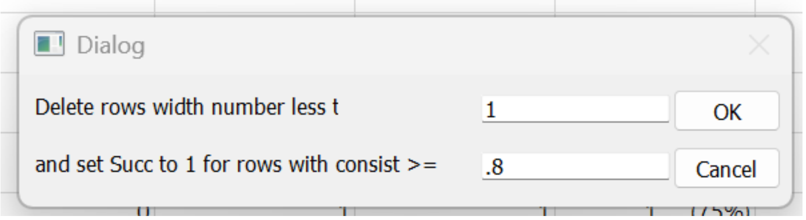

- Compute; inspect contradictory configurations (rows with similar membership but different outcomes).

IntFuzz	ExtFuzz	IntegBur	OpTrain	Mgmt	IntAgen	PubEdTim	PubEdVis	Endor	MedCov	number	Succ	cases	raw consi	PRI consis	SYM consist
0	1	1	1	1	1	0	1	1	1	2	1		1	1	1
1	0	0	1	1	1	1	0	0	1	1	1		1	1	1
0	1	1	1	1	1	0	1	0	1	1	1		1	1	1
0	1	1	1	1	1	0	0	1	1	1	1		1	1	1
1	0	0	1	1	1	1	1	1	1	1	1		1	1	1
1	0	1	1	1	1	1	1	1	1	1	1		1	1	1
0	1	1	1	1	1	1	0	0	1	1	1		0.953132	0.953132	0.953132
0	1	1	1	1	1	1	0	1	1	1	1		0.947642	0.947642	0.947642
0	0	1	1	1	1	1	0	1	1	1	0		0.008142	0.008142	0.008142
0	0	0	0	1	1	0	0	0	0	1	0		0.002531	0.002531	0.002531
0	0	1	0	1	1	0	0	0	1	1	0		0.002531	0.002531	0.002531
0	0	0	0	0	0	0	0	0	0	1	0		0	0	0
0	0	1	0	0	1	0	0	0	0	1	0		0	0	0
0	0	1	1	0	0	0	0	0	1	1	0		0	0	0
0	0	0	0	0	0	1	0	0	1	1	0		0	0	0

- Verify raw consistency (i.e., proportion of cases that fall into a specific causal configuration and have the outcome, relative to the total number of cases with that outcome) and PRI consistency (i.e., measures how well a specific causal combination explains the outcome, representing the degree to which the cases that have the combination also have the outcome, relative to cases that do not have the outcome but have the combination). Typically, raw consistency and PRI consistency scores should be > 0.8 and close to each other for relevance and inclusion of truth table rows in the QCA solution.[9]

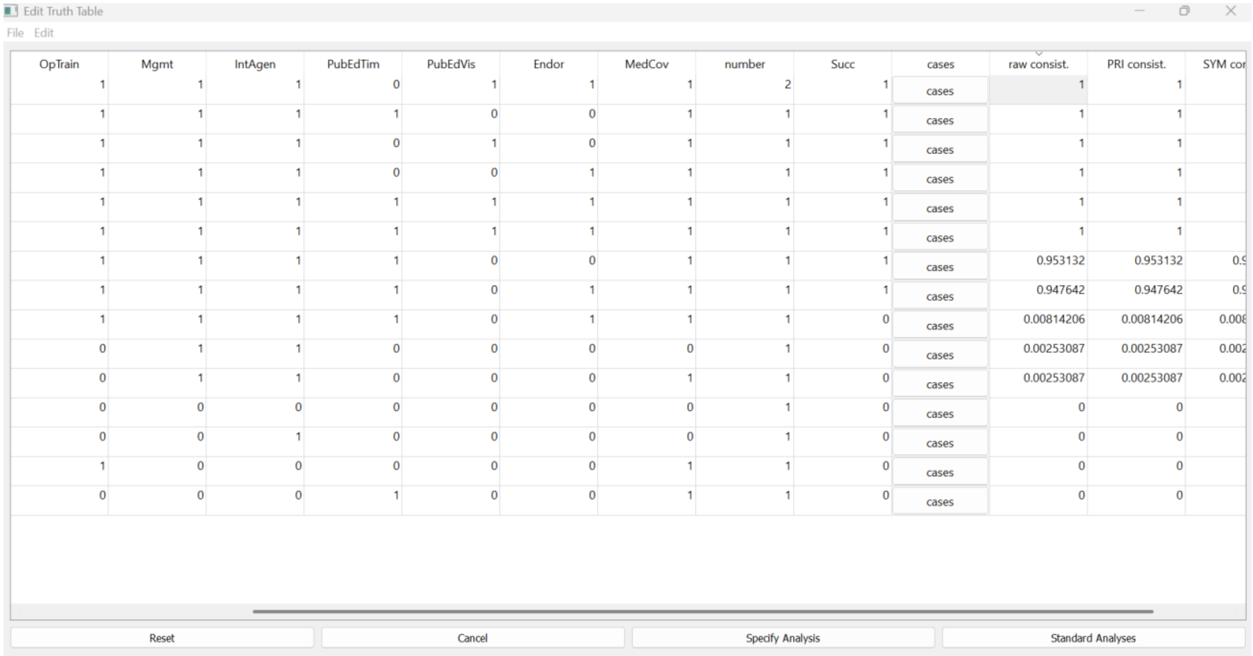

OpTrain	Mgmt	IntAgen	PubEdTim	PubEdVis	Endor	MedCov	number	Succ	cases	raw consist.	PRI consist.	SYM cor
1	1	1	0	1	1	1	2	1	cases	1	1	
1	1	1	1	0	0	1	1	1	cases	1	1	
1	1	1	0	1	0	1	1	1	cases	1	1	
1	1	1	0	0	1	1	1	1	cases	1	1	
1	1	1	1	1	1	1	1	1	cases	1	1	
1	1	1	1	1	1	1	1	1	cases	1	1	
1	1	1	1	0	0	1	1	1	cases	0.953132	0.953132	0.9
1	1	1	1	0	1	1	1	1	cases	0.947642	0.947642	0.9
1	1	1	1	0	1	1	1	0	cases	0.00814206	0.00814206	0.008
0	1	1	0	0	0	0	1	0	cases	0.00253087	0.00253087	0.002
0	1	1	0	0	0	1	1	0	cases	0.00253087	0.00253087	0.002
0	0	0	0	0	0	0	1	0	cases	0	0	
0	0	1	0	0	0	0	1	0	cases	0	0	
1	0	0	0	0	0	1	1	0	cases	0	0	
0	0	0	1	0	0	1	1	0	cases	0	0	

Table S13. Truth table generated after thresholds for minimum number of cases with success outcome (=1) and consistency frequency (=0.80) were specified.

IntAgen	PubEdTim	PubEdVis	Endor	MedCov	IntFuzz	ExtFuzz	InteгBur	OpTrain	Mgmt	number	Succ	raw consist.	PRI consist.
1	0	1	1	1	0	1	1	1	1	2	1	1	1
1	1	0	0	1	1	0	0	1	1	1	1	1	1
1	0	1	0	1	0	1	1	1	1	1	1	1	1
1	0	0	1	1	0	1	1	1	1	1	1	1	1
1	1	1	1	1	1	0	0	1	1	1	1	1	1
1	1	1	1	1	1	0	1	1	1	1	1	1	1
1	1	0	0	1	0	1	1	1	1	1	1	0.95	0.95
1	1	0	1	1	0	1	1	1	1	1	1	0.95	0.95
1	1	0	1	1	0	0	1	1	1	1	0	0.01	0.01
1	0	0	0	0	0	0	0	0	1	1	0	0.00	0.00
1	0	0	0	1	0	0	1	0	1	1	0	0.00	0.00
0	0	0	0	0	0	0	0	0	0	1	0	0	0

1	0	0	0	0	0	0	1	0	0	1	0	0	0
0	0	0	0	1	0	0	1	1	0	1	0	0	0
0	1	0	0	1	0	0	0	0	0	1	0	0	0

Key: IntAgen = *Committed Interagency Agreements*, PubEdTim = *Public Education ≥ 24 Months Pre-decision*, PubEdVis = *Permanent Demo/Visitor Center*, Endor = *Local Endorsement from Community Spokespersons*, MedCov = Positive Media Coverage, IntFuzz = Sufficient Internal/Private CAPEX Funding, ExtFuzz = Sufficient External Public CAPEX Funding, IntegBur = *Low Infrastructure Integration Burden*, OpTrain = *Sufficient Operator Training*, Mgmt = *Continuity in Project Leadership*, number = Number of Cases, Succ = Successful, raw consist = Raw Consistency of Each Truth Table Row, PRI consist = Proportiional Reduction in Inconsistency of Each Truth Table Row

vii. Analyze → Standard Analysis:

- Select "Standard Analyses" to identify all three solution types (i.e., complex, parsimonious, and intermediate).[9] We report the intermediate solution for this analysis.
- Solution types:
  i. Complex (no logical remainders used)
  ii. Parsimonious (all remainders allowed, regardless of directional expectations (DEs); see below for an explanation of DEs)
  iii. Intermediate (remainders consistent with DEs only; primary solution we report)

Step 5. Specify DEs for intermediate solutions

viii. Assumptions for DEs: Set which conditions are expected to be positively (↑) or negatively (↓) associated with Y based on theory and empirical priors.

- We hypothesized the *presence* of each causal condition to be *positively* associated with success.

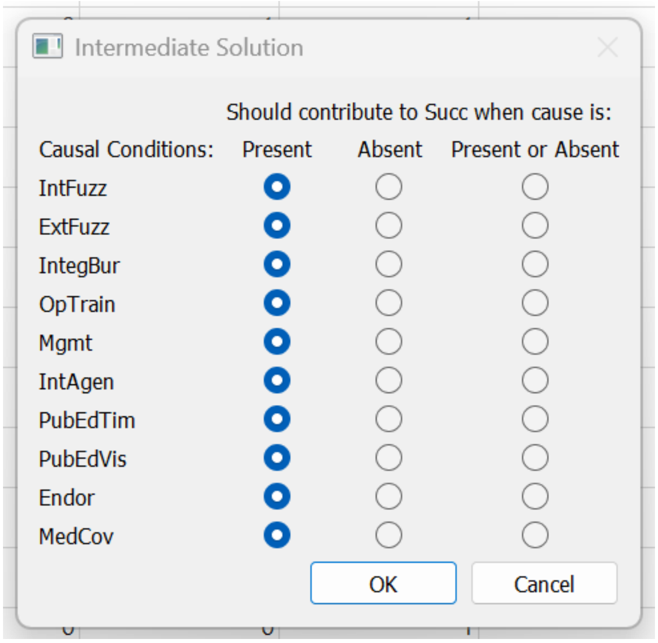

ix. NOTE: A Prime Implicant Chart appeared, for which we chose “Select All” since Prime Implicants are used to compute the complex solution, which was not the solution of interest because complex solutions mirror every observed conjunction of causal conditions, regardless of case knowledge via incorporation of DEs. Since DEs are explicit and justify which remainders we used; our results truly highlight the more portable pathways than complex solution pathways.

Step 6. Minimize truth table (derive solutions)

- Run. Export solutions.

```
--- INTERMEDIATE SOLUTION ---
frequency cutoff: 1
consistency cutoff: 0.947642
Assumptions:
IntFuzz (present)
ExtFuzz (present)
IntegBur (present)
OpTrain (present)
Mgmt (present)
IntAgen (present)
PubEdTim (present)
PubEdVis (present)
Endor (present)
MedCov (present)
                                                           raw       unique
                                                         coverage    coverage   consistency
                                                        ----------  ----------  ----------
IntFuzz*OpTrain*Mgmt*IntAgen*PubEdTim*MedCov            0.306022    0.211433    0.976805
ExtFuzz*IntegBur*OpTrain*Mgmt*IntAgen*PubEdTim*MedCov   0.307456    0.0377778   0.976911
ExtFuzz*IntegBur*OpTrain*Mgmt*IntAgen*PubEdVis*MedCov   0.235233    0.0377778   1
ExtFuzz*IntegBur*OpTrain*Mgmt*IntAgen*Endor*MedCov      0.383011    0.0377778   0.981381
solution coverage: 0.67
solution consistency: 0.989271


Cases with greater than 0.5 membership in term IntFuzz*OpTrain*Mgmt*IntAgen*PubEdTim*MedCov: 1 (0.67,1),
  3 (0.67,1), 8 (0.67,1)
Cases with greater than 0.5 membership in term ExtFuzz*IntegBur*OpTrain*Mgmt*IntAgen*PubEdTim*MedCov: 4 (0.67,1),
  5 (0.67,1)
Cases with greater than 0.5 membership in term ExtFuzz*IntegBur*OpTrain*Mgmt*IntAgen*PubEdVis*MedCov: 2 (0.67,1),
  6 (0.67,1), 9 (0.67,1)
Cases with greater than 0.5 membership in term ExtFuzz*IntegBur*OpTrain*Mgmt*IntAgen*Endor*MedCov: 2 (0.67,1),
  4 (0.67,1), 7 (0.67,1), 9 (0.67,1)
```

x. Record for each solution and pathway:
- Solution expression/pathway (e.g., IntFuzz*OpTrain*Mgmt*IntAgen*PubEdTim*MedCov)
- Overall model fit: solution consistency (here, 0.99) and solution coverage (here, 0.67)
- For each solution pathway: raw coverage, unique coverage, and consistency

<u>S4.3.2. Sensitivity Analysis</u>

S4.3.2.1 Cutoff sensitivity: Re-run Standard Analysis varying:

Consistency (truth table): 0.75 and 0.85. We found that results remained unchanged.

0.75:

```
--- INTERMEDIATE SOLUTION ---
frequency cutoff: 1
consistency cutoff: 0.947642
Assumptions:
IntFuzz (present)
ExtFuzz (present)
IntegBur (present)
OpTrain (present)
Mgmt (present)
IntAgen (present)
PubEdTim (present)
PubEdVis (present)
Endor (present)
MedCov (present)
                                                           raw       unique
                                                         coverage   coverage   consistency
                                                        ---------- ---------- ----------
IntFuzz*OpTrain*Mgmt*IntAgen*PubEdTim*MedCov            0.306022   0.211433   0.976805
ExtFuzz*IntegBur*OpTrain*Mgmt*IntAgen*PubEdTim*MedCov   0.307456   0.0377778  0.976911
ExtFuzz*IntegBur*OpTrain*Mgmt*IntAgen*PubEdVis*MedCov   0.235233   0.0377778  1
ExtFuzz*IntegBur*OpTrain*Mgmt*IntAgen*Endor*MedCov      0.383011   0.0377778  0.981381
solution coverage: 0.67
solution consistency: 0.989271


Cases with greater than 0.5 membership in term IntFuzz*OpTrain*Mgmt*IntAgen*PubEdTim*MedCov: 1 (0.67,1),
  3 (0.67,1), 8 (0.67,1)
Cases with greater than 0.5 membership in term ExtFuzz*IntegBur*OpTrain*Mgmt*IntAgen*PubEdTim*MedCov: 4 (0.67,1),
  5 (0.67,1)
Cases with greater than 0.5 membership in term ExtFuzz*IntegBur*OpTrain*Mgmt*IntAgen*PubEdVis*MedCov: 2 (0.67,1),
  6 (0.67,1), 9 (0.67,1)
Cases with greater than 0.5 membership in term ExtFuzz*IntegBur*OpTrain*Mgmt*IntAgen*Endor*MedCov: 2 (0.67,1),
  4 (0.67,1), 7 (0.67,1), 9 (0.67,1)
```

0.85:

```
--- INTERMEDIATE SOLUTION ---
frequency cutoff: 1
consistency cutoff: 0.947642
Assumptions:
IntFuzz (present)
ExtFuzz (present)
IntegBur (present)
OpTrain (present)
Mgmt (present)
IntAgen (present)
PubEdTim (present)
PubEdVis (present)
Endor (present)
MedCov (present)
                                                           raw       unique
                                                         coverage   coverage   consistency
                                                        ---------- ---------- ----------
IntFuzz*OpTrain*Mgmt*IntAgen*PubEdTim*MedCov            0.306022   0.211433   0.976805
ExtFuzz*IntegBur*OpTrain*Mgmt*IntAgen*PubEdTim*MedCov   0.307456   0.0377778  0.976911
ExtFuzz*IntegBur*OpTrain*Mgmt*IntAgen*PubEdVis*MedCov   0.235233   0.0377778  1
ExtFuzz*IntegBur*OpTrain*Mgmt*IntAgen*Endor*MedCov      0.383011   0.0377778  0.981381
solution coverage: 0.67
solution consistency: 0.989271


Cases with greater than 0.5 membership in term IntFuzz*OpTrain*Mgmt*IntAgen*PubEdTim*MedCov: 1 (0.67,1),
  3 (0.67,1), 8 (0.67,1)
Cases with greater than 0.5 membership in term ExtFuzz*IntegBur*OpTrain*Mgmt*IntAgen*PubEdTim*MedCov: 4 (0.67,1),
  5 (0.67,1)
Cases with greater than 0.5 membership in term ExtFuzz*IntegBur*OpTrain*Mgmt*IntAgen*PubEdVis*MedCov: 2 (0.67,1),
  6 (0.67,1), 9 (0.67,1)
Cases with greater than 0.5 membership in term ExtFuzz*IntegBur*OpTrain*Mgmt*IntAgen*Endor*MedCov: 2 (0.67,1),
  4 (0.67,1), 7 (0.67,1), 9 (0.67,1)
```

S4.3.2.2 Subset/Superset Analysis

In set-theoretic terms, subset/superset analysis checks whether one condition-set is *contained within* (subset of) or *contains* (superset of) another condition-set. This is used as a redundancy/collinearity diagnostic prior to minimization: if X_i is (almost) a subset of X_j (i.e., wherever cases belong to X_i they also belong to X_j), then X_i adds little distinct information and can be considered for removal or re-specification.[4,9,10] Subset/superset analysis is informative when candidate conditions overlap to some degree (e.g., two partially overlapping competencies, two related institutional arrangements). It helps avoid double-counting near-duplicates, clarifies hierarchical relations among conditions, and can motivate combining or dropping a condition prior to truth table construction.

We ran pairwise subset tests among all calibrated condition-sets included in the primary model. The only relation that mechanically appeared removable was *Internal/Private CAPEX Funding* vis-à-vis *External Public CAPEX Funding*. However, in our dataset funding sets are encoded to be mutually exclusive by design:

- Each case is assigned to exactly one predominant funding source;
- Consequently, the fuzzy memberships for *internal* and *external* are disjoint (in fuzzy membership, they are constrained not to co-rise and typically sum to ≈1).

Under such disjoint encodings (i.e., sets are mutually exclusive), subset indices become uninformative because the two sets are constructed to be complements/alternatives rather than overlapping constructs. Because the apparent removability of *Internal/Private CAPEX Funding* arises solely from the coding scheme rather than from a substantive dominance relation, we judged the subset/superset statistics to not be meaningful for these funding causal conditions and did not drop either funding construct on this basis. For all other pairs where overlap was possible,

subset/superset checks showed no high-inclusion redundancy (i.e., similar solution consistency but better solution coverage), so we retained the specified causal conditions.

S4. Case Summaries

All procedures followed the latest STROBE guidance for observational studies. Before participation, interviewees received an information sheet describing purpose, risks, voluntariness, and data handling; verbal consent was obtained prior to recording. Individually identifying details were removed during transcription; de-identification keys were stored separately on an encrypted hard disk. Field activities occurred in active water/wastewater facilities and, in some cases, construction zones. We followed host-utility safety protocols and escorted-access requirements. No extraordinary hazards beyond those typical utility facilities were encountered. Interviews posed minimal risk, and participants could cease participation at any time.

Table S14. Dictionary of acronyms used.

Acronym	*Full Form*	*Definition*	*Mentioned in Case ___*
12-10-10 Rule	--------------------------	A disinfection-level rule, dictating 12-log removal of viruses, 10-log of *Cryptosporidium*, and 10-log of *Giardia*	4, 6, 12
AFY (or AF)	Acre-Feet per Year (or Acre-Feet)	A unit of volume per time, used by water agencies when talking about large quantities of water	6, 9
AMTA	American Membrane Technology Association	An organization dedicated to promoting and advancing the understanding and application of membrane technologies in water treatment contexts	10
AOP	Advanced Oxidation Process	Any tertiary water treatment process that involves generating hydroxyl radicals to breakdown contaminants into non-toxic byproducts	2, 4, 8, 12
ATF	Advanced Treatment Facility	A water-processing site that preforms advanced water treatment (AWT)	9
AWP	Advanced Water Purification	Another name for Advanced Water Treatment, varies by the region	8, 16
AWS	Alternative Water Supplies	Non-traditional water sources aimed and sustainably supplementing conventional freshwater sources (e.g. rivers, groundwater)	3
AWT	Advanced Water Treatment	A broad delimitation of tertiary steps in water treatment processes	5, 8, 12, 16
AWTO	Advanced Water Treatment Operator	A general label for operators who are certified to oversee advanced water treatment (AWT) processes	9

CapEx	Capital Expenditure	In the world of finance, CapEx is the funds investing in physical assets like materials and land	4
CCP	Critical Control Point	Any specific step in the water treatment process that, if managed improperly, will lead to human health risks	2, 9, 12
CEC	Contaminants of Emerging Concern	Any water contaminant that in not well understood but is becoming increasingly apparent in water samples	4, 9, 15
CIP	Capital Improvement Program	A long-term plan that acts as a timeline for project capital needs and investments; a fortified budget	12
CMMS	Computerized Maintenance Management System	A water utility software that helps track and manage maintenance on infrastructure and physical assets	6
DPR	Direct Potable Reuse	The process of injecting highly treated municipal wastewater directly into drinking-water supply (opposed to IPR)	1, 3, 7, 8, 10, 11, 14, 16
FAQ	Facts and Questions	Usually, a source of information and commonly asked questions presented to the public in an attempt to streamline addressing common concerns	7
FTE	Full-Time Equivalent	A unit of measurement, representing one full-time (24/7) employee's worth of workload and staffing needs (e.g. how many new staff would be needed to fulfill the requirement of one hypothetical 24/7 employee)	14
GAC	Granular Activated Carbon	GAC is a form of carbon with a large surface area, created by processing materials like coconut shells, coal, or wood through heating and activation to create a porous structure enabling it to adsorb contaminants from liquids and gases as they pass through its pores.	4, 5, 10, 12, 13, 15
GPM	Gallons Per Minute	A unit of volume per time, used in a variety of fluid flow-rate applications	4,16
HAB	Harmful Algal Bloom	An over-growth of algae or cyanobacteria in a water supply which produce toxins harmful to animals and humans, and alter water tase and odor	5
HDPE	High-Density Polyethylene	A strong thermo-plastic polymer. Used in pipes for fluid transport, especially wastewater and industrial fluids	7
IPR	Indirect Potable Reuse	The process of buffering treated municipal wastewater with a natural body of water (opposed to DPR)	1,3,4,5,7,8,11,12,14,15
ISAP	Independent Scientific Advisory Panel	A panel of qualified experts who provide advice and suggestions objectively	12

KPI	Key Performance Indicator	A measurable value that tracks an entity's performance against its goals	4,12
MAR	Managed Aquifer Recharge	An intentional injection of "clean" surface water into aquifers, managing water supply, subsidence, and saltwater intrusion	2,6,2
MBR	Membrane Bio-Reactor	A secondary and/or tertiary water treatment processes that involves a combination of membrane filtration and a biological treatment (activated sludge)	10
MF	Microfiltration	A tertiary water treatment process that involves forcing water through membranes, which filter out contaminants as small as 0.1 to 10 microns (μm) (e.g. bacteria) (similar to UF, uses less pressure)	4,7,9,16
MGD	Million Gallons per Day	A common unit for measuring flow rates in water sources	2,3,4,6,8,12,14
MOC	Membrane Operator Certification	An advanced credential for professional water treatment operators specializing in membrane processes	10
NDMA	N-Nitrosodimethylamine	A disinfection by-product that is a known, probable human carcinogen	6,9,12
NGO	Non-Governmental Organization	A not-for-profit organization that is not associated with the government, usually tackling political or social issues	12
NPDES	National Pollutant Discharge Elimination System	A federal permitting program that regulates point-source discharging into US waters	6
O&M	Operations and Maintenance	The phase after project delivery that involves upkeep, management, and improvement of the facility	1,2,3,4,5,6,7,8,10,11,12,13,14,15
PER	Preliminary Engineering Report	A document that reviews the economic, technical, and environmental feasibility of a project to advise the final design	10
PFAS	Polyfluoroalkyl Substances	Chemical compounds that are hard to breakdown naturally, which can contaminate water sources and cause bioaccumulation	2, 4, 12, 15, 16
QA	Quality Assurance	A final "check" of a sample or few samples to assure quality (as opposed to continual inspection through quality control)	10, 13
RO	Reverse Osmosis	A tertiary water treatment process that involves forcing water through a semi-permeable membrane to separate "permeate" (freshwater) and "brine" (residual)	4,6,7,8,9,10,12,16
SCADA	Supervisory Control and Data Acquisition	A monitoring system that allows operators to see and control treatment-system parameters remotely	5,7,16

SOP	Standard Operating Procedure	Set of step-by-step instructions that detail how to perform a certain task	3
SRF	State Revolving Fund	A state-level financing for infrastructure projects, such as drinking water, wastewater, and stormwater	7,9,15
SWS	Sensitive Water Supply	A source of water that is particularly prone to contamination, usually due to the nature of its location (e.g. surface water, well water, etc.)	15
TMDL	Total Maximum Daily Load	The maximum amount of contaminant a water source can hold before it surpasses standards set by the Clean Water Act	15
TMF	Technical, Managerial, and Financial	A group of subject-areas that are necessary for long-term project success (usually discussed as "TMF capacity")	10
TOC	Total Organic Carbon	A measurement of all organic matter in a water sample, tracked through the total mass of Carbon in a sample	4,5,9,12
TSS	Total Suspended Solids	The mass per liter of suspended, non-dissolved particles found in water sources, usually addressed by filters	5
UF	Ultrafiltration	A tertiary water treatment process that involves forcing water through membranes, which filter out contaminants as small as 0.001 to 0.1 microns (µm) (e.g. bacteria, viruses) (similar to MF, more pressure)	4,6,8,10,16
UIC	Underground Injection Control	An EPA program established under the Safe Drinking Water Act that regulates deep-well injection to protect groundwater resources	2
UV	Ultraviolet	A wavelength of light that is used for inactivating pathogens so they cannot reproduce and cause harm to humans	7,9,10,12,15
UV-AOP	Ultraviolet Advanced Oxidation Process	A tertiary water treatment process that involves UV-activation of oxidants into hydroxyl radicals, which break down contaminants into non-toxic byproducts	4,5,6,7,8,9,10,13,14,15,16
VR	Virtual Reality	A technological innovation that allows users to explore a virtual world in a realistic way, utilizing headsets and handheld controls to navigate	8,16
WIFIA	Water Infrastructure Finance and Innovation Act	A government program that gives low-cost loans to projects involving water infrastructure (e.g. WWTP, etc.)	13,14
WWTP	Wastewater Treatment Plant	Facilities (either municipal or private) that treat residential and/or industrial wastewater for reuse or safe discharge into the environment	10

Case 1

Implementation outcome: Successful

Driver: Severe drought in early-2000s, drinking water reservoir drying up

Method: IPR project with anticipation to transition to DPR, return flows intercepted downstream, natural purification then advanced purification integrated in existing distribution infrastructure, multibarrier design, biologically active carbon & advanced oxidation, integrated program management model

Results: Added 10,000 acre-feet of water per year at startup, permanent pilot established for testing, built social license to pave future DPR projects

Details:

- Case completed ahead of schedule (design to first water in 5 years), remained well under budget, sized for growth with plans to expand
- No initial specific state reuse regulations, so health agency stepped in
  - Permitted facility as conventional plant with additional barriers
- Pilot used to optimize the process and provide regulation-readiness in anticipation of a future transition to DPR
- Utility voluntarily committed to a comprehensive outreach plan
  - Early and ongoing outreach branded with accessible language, included schools, managed facility tours, etc.
  - Logged over 100 public events
  - Citizen body marginally involved in decision-making
- Financed \$653–\$637 million with local municipal bonds, connection fees, rate revenues, a low-interest state loan (~12%) from the water conservation board, and rigorous value engineering under an integrated program management model
  - O&M factored into the broader enterprise's debt-service and operations portfolio, assures long-term financing
- Integrated program management model was used to streamline and consolidate projects and tasks (based out of a shared digital platform)
  - Utility negotiated across jurisdictions for 400+ permits without slipping schedule
- Operators were hired before construction to witness and understand the whole process and were taught through custom and expert-developed curriculum
- Project also relied on cross-jurisdictional regulatory workplans and delivery/purchase-style contracts that reduced permitting and operational uncertainty

Case 2

Implementation outcome: Successful

Driver: Compliance with regional groundwater and surface water regulations, fend off saltwater intrusions and slow down land subsidence (ground sinking)

Method: Used MAR wells (Class V deep well injection of secondary effluent after a carbon-based AOP)

Results: ~16 MGD treatment facility under construction with future capacity of 30 MGD, 1 MGD demonstration facility for research and education, institutionalized regulatory collaboration

Details:

- Need for compliance with local regulations propelled utility to consider a water reuse program, multiple benefits included recharging an aquifer, staving off saltwater intrusion, and slowing subsidence
- Project was operated under the federal Underground Injection Control (UIC) and a state-level oversight committee, assuring public transparency during project ramp-up
- Permits directed activities (e.g. CCP, language for monitoring new contaminants) and dictated standards for operation (e.g. injection volume and pressure), creating a clear pathway for implementation
- Tight compliance deadlines drove efficient scheduling and intense construction timelines
- Avoided "pilot-trap" because the "pilot" was a permanent demonstration platform
- Capital dominated by federal loans (~45% of costs) and low-interest state loans (the rest, mostly) plus revenue bonds
    - Federal loan agreement phased multiple loans as projects reached certain deliverables
    - O&M funded through utility rate revenues, absorbed into existing enterprise
- Reuse-plus-nutrient strategy (instead of linear sewer reconstruction) heavily benefitted the environmental scene by replenishing groundwater and recycling nutrients
    - Reuse instead of conveyance rebuilds continually cited as the most cost-effective path to meet goals
- Tour-centric demonstration "pilot"/center, persistent and professional public communications, and technical reports garnered public attention
    - Legislated public-oversight committee regularly published reports on technical progress (highlighting its dual nature of demonstration and research facility)
    - Operators trained on exact full-scale process from the get-go at the pilot
- Success built around external public financing (federal + state loans) paired with institutionalized oversight and interagency coordination that clarified roles and compliance obligations

Case 3

Implementation outcome: Successful

Driver: Long-range analysis showed peak season supply gaps & utility's consumptive-use permit was up for renewal anyways, state's "eliminate non-beneficial surface discharges" statute

Method: IPR (with potential for later DPR)

Results: Two full-scale treatment facilities (first 10 MGD, expandable to 20 MGD)

Details:

- Long-term projections showed that current water supplies would not be sufficient for peak-season demand
- Utility's consumptive-use permit was up for renewal in a few years
- State issued a potable reuse rule in 2025 which required utilities to submit elimination plans (for non-beneficial surface discharge) and fully implement them by 2032
- Implementation sequence: pilot → 1-MGD demonstration, visitor center → two full-scale advanced treatment facilities totaling ~10–20 MGD
    - Pilot and demo user-rate funded (utility capital and bonds)
- "Minimize discharge with potable reuse" paths dominated the cost-benefit rankings
- Tour-centric demonstration center has tasting area and curriculum co-developed with regional partners (aimed at garnering acceptance or neutrality over overtly positive attitudes)
- Public decision-making was mostly informative (briefings and listening sessions)
- Operator hiring opportunities were integrated into the demo center (fulfilling the need for staff in this "new line of business"), state-wide trainings and certificates were developed with professional associations, operators experienced with demo center operations
    - O&M funded completely through the utilities operating budget
- Utility met (bi-weekly) with state environmental-regulators, coordinated on the potable reuse framework efforts, and internally helped cross-department meetings to reduce organizational friction

Case 4

Implementation outcome: Successful

Driver: A long-standing supply deficit forced a moratorium/block on new connections to avoid overloading the existing water infrastructure

Method: IPR project, tertiary effluent goes through MF/UF → RO → UV-AOP → biologically active carbon, then conveyed ~7 miles to a surface reservoir (buffer) where finished water is dechlorinated prior to mixing

Results: 0.5 MGD IPR plant

Details:

- Small and expandable advanced purification facility (sends purified water to off-site surface reservoir as a buffer, then performs conventional treatment)
- "Final" product was an upgrade from a 20 GPM pilot that served as a testing center:
    - Tested MF and UF pretreatment
    - Validated two-stage RO behavior at ~75–80 % recovery
    - Used UV-AOP dosing for 1,4-dioxane control
    - Demonstrated post-AOP GAC for peroxide polishing and TOC step-down
    - Reported non-detects/low detect for a broad CEC/PFAS scan
    - Provided evidence that GAC on RO-concentrate stream reduces PFAS levels
- Above test results helped establish KPI-based diversion logic for sub-par/off-spec water
- Policy/regulation established a pilot program that explicitly authorized potable reuse permits (with public participation) and detailed tech requirements
    - Effluent must meet or surpass drinking water standards, uphold 12/10/10 pathogen-reduction targets across all stages of treatment, utilize an environmental-buffer residence time (generally ≥ 180 days), and incorporate RO and UV disinfection at relative stages
- High infrastructure readiness (conveyance corridor already existed; the IPR site and reservoir interface were defined)
- Capital: 2/3 external grants (~68%), 1/3 internal funding (~32%)
- Public education and awareness involved branding, websites, and surveys
- Interagency collab involved state environment departments, universities, national research organizations, peer utilities, and technology suppliers
- Operators built and ran the 9-month pilot, and were heavily involved in decision-making

Case 5

Implementation outcome: Successful

Driver: Recurring drought put pressure on a shared reservoir, prompting legal disputes and a search for new, locally controlled supply

Method: Pilot used microfiltration and media filtration followed by GAC and UV-AOP

Results: In-progress full-scale implementation, first-of-its-kind project funded and approved by the state, qualified operation and maintenance staff

Details:

- Persistent supply stress was exposed during an early-2000s and subsequent droughts
- State's codified IPR rule laid out a six-step ladder (feasibility, pilot if required, wasteload allocation/modeling, engineering report, permit application with contingency plan, and final design) and prescribed multi-barrier performance, conservative operation benchmarks, HAB-response requirements, and dual Class-A certifications for responsible operators
    - The utility aligned its planning to these frameworks
- A multi-month field pilot (used a carbon-based advanced-treatment train to avoid brine production) submitted its results for state review
    - Operators and partners ran the pilot and documented data/performance, which directed SCADA and control priorities for the full-scale project
- Infrastructure readiness was improved by a pre-built conveyance pump station and a several-mile pipeline to the augmentation point, funded by a time-limited grant milestone
- The full-scale project was financed almost entirely by state loans (~89%) and federal drought resiliency grants (~11%)
- Public outreach was minimal, involving pilot tours by invitation and public briefings over multiple years, yet public sentiment was largely supportive and only had a small opposing minority online
- Public education was absent, but a standing committee was decisive in approving the project
- Coordination with multiple agencies (state regulators, federal partners, and external technical teams) was present through pilot and design development, regulators were supportive yet required robust documentation for the first-of-its-kind project
- Operators were crucial in the project (ran the pilot, on track to obtain required certifications, and identified operational cost drivers that shaped O&M to seasonal differences)

Case 6

Implementation outcome: Successful

Driver: Supply volatility sparked a need for climate-resiliency in water supplies, long history of relying on distant aqueducts

Method: UF --> RO --> UV-AOP designed with racks, stages, and the oxidation step sized for constituents of concern (1,4-dioxane and NDMA), waste to be applied to spreading grounds or discharged into a river channel when grounds were unavailable

Results: Produced ~14.8 MGD of purified water and maintained an objective to supply at minimum ~10,000 AFY for MAR

Details:

- Climate resiliency of local water resources was the main goal of a basin-wide independence program (to protect against persistent drought and supply volatility)
- The final facility produced up to ~14.8 MGD of purified water with a minimum annual production objective of ~10,000 AFY for managed aquifer recharge
- The purification project operated under two permits (required to meet the most stringent conditions applicable at each discharge point):
    - waste discharge-reuse order for deliveries to spreading grounds (in wet years, the project throttled production for a few months until hydraulic space returned)
    - NPDES permit for controlled discharge to a river channel (when spreading capacity was reached)
- The MAR part of the project was required to meet the 12-10-10 pathogen log-reduction performance (which was monitored and documented)
- The project was developed under a design-build-operate delivery, where a 30-day continuous run was required before project hand-off
- Flexibility between spreading or discharging allowed extended testing and acceptance without compromising compliance
- The project was financed through a mix of external and internal funding (final capital costs were on the order of $134 M)
    - all-in delivered-water cost (~$1,474/AF) converged toward untreated imports
- A contract-operations model under a multi-year agreement covered the agency's two advanced treatment facilities, and supported a mature operator pathway for AWT (covered by the utility's rate base and heavily offset by state loan funding)
- Public education was a permanent core function of the plant, curating a museum and gardens for people of all languages to learn about reuse, in addition to other efforts
    - A permanent public-facing education function (museum-style learning environment) normalized potable reuse while also supporting operator pathway maturity through structured, hands-on exposure
- Utility depended on close coordination with regional water-quality regulators and the flood-control/watershed manager for permit approvals and spreading-ground & river operations, respectively

Case 7

Implementation outcome: Successful

Driver: Extreme drought affecting two primary reservoirs, pushing them to critically low storage, emergency response required

Method: Expedited DPR (MF → RO → UV-AOP → blend with raw surface water), post-emergency IPR (cloth media and phosphorus removal → lake diffusers)

Results: Long-term IPR system as drought insurance

Details:

- Multi-year drought pushed water utility into an emergency as water supplies dwindled
- State's established potable reuse program allowed for an expedited DPR project in this situation, as it approved advanced treatment on a case-by-case basis under its "innovative/exception" authority
- Project (both in the emergency phase and long-term phase) was navigated under intensive oversight from the state
- Emergency Phase (DPR):
    - Large-diameter HDPE line conveyed secondary effluent to an advanced treatment skid (MF → RO → UV-disinfection/advanced oxidation), then blended the purified stream with raw surface water before conventional treatment
    - Public outreach included regular communications and tastings, engagement with health professionals. Feedback favored taste/odor from the blended supply
    - Funded through local enterprise financing
- Long-term Phase (IPR):
    - Repurposed the same conveyance system for tertiary effluent (polished with cloth media and phosphorus removal) to mid-lake diffusers designed to achieve detention prior to diversion and conventional treatment
    - Public communication and participation continued with public notice and hearing processes (enabling public procedural participation)
    - Used SRF assistance (loan in the mid-30-million range, funded 99+% of IPR)
    - Users supported rate-hike to repay above debt
- O&M treated as a whole-enterprise function, with operators integrating advanced treatment run rules with conventional-plant hydraulics and corrosion control
    - Funded through user fees/water rates
- Operators in drinking water and wastewater divisions cross-trained during emergency phase, with timely monitoring and audits, supported maintenance expertise
- Interagency collaboration and external support was paramount in both phases

Case 8

Implementation outcome: Successful

Driver: Stressed surface water imports needed a defense against drought (DPR), IPR project had already been operational for ~3 decades

Method: Pre-existing IPR project (UF → cartridge → RO → UV-AOP → lime decarbonation), proposed DPR project (need to add AOP/control and monitoring upgrades)

Results: DPR project on-track (hinging on filling operator gaps)

Details:

- Community was familiar with AWT due to a longstanding (~3 decade) IPR project (produced nominally around 20 MGD), DPR upgrade/addition was being pursued to secure water resources during periods of drought that stressed surface water imports
  - Water treatment utility was well-prepared for a pivot to DPR due to a diverse water-resource portfolio (raw water pumping, vadose-zone wells, and a reclaimed water distribution grid)
  - Large landscape customers had priority access to reclaimed non-potable water, therefore careful waiver management was necessary for supplying DPR operations without squeezing that market
- Infrastructure readiness was high, only upgrades to a pre-existing IPR project were needed, concentrate management and solids handling were already solved within regional systems (reduced implementation risk)
- A state AWP rule package mandated program elements and AWP operator certification
  - Program elements included multibarrier treatment expectations, critical control points, and monitoring tiers
  - Operator requirements included a new licensing category exceeding conventional treatment licensing (field-certification at 0.5MDG pilot), proved to be a barrier to workforce growth due to the need for long daily presence of certified personnel who were few in number
- Initial IPR project was 100% city-funded (municipal bonds and development fees), O&M was paid for by water rates, funding for DPR upgrade (tens of millions) was not fully appropriated (need for federal loans or SRF)
- Approachable language, such as "purified recycled water," was used in public outreach which scored potable reuse ~70% acceptance once people saw the treatment and science for themselves
  - Utility ran 40+ tours per year of IPR project, VR content, used brewery showcases to normalize the concept of potable reuse, conducted onsite water tastings
- Implementation capacity was strengthened through private/internal financing, including explicit commitments with private developers for capital/O&M in exchange for priority deliveries, alongside clear regulatory coordination
- Full-scale implementation progress on-track

Case 9

Implementation outcome: Successful

Driver: An acute water-supply constraint, due to regulations, created the need for reuse

Method: MAR after a membrane-based, RO-centric multi-barrier process with defined log-reduction credits and a response-retention-time concept anchored by subsurface travel times and continuous treatment monitoring

Results: AWT facility for MAR into coastal aquifers

Details:

- Regulations limited river diversions, so reuse/injection was a way of reducing surface-water extraction by capitalizing on aquifer resources through MAR
  - MAR performed on the order of several thousand AFY through clusters of deep-zone and vadose-zone injection wells, with an additional drought-reserve banking feature for extra injection in wet years
  - AWT through four-barrier train: ozone pretreatment, membrane filtration, reverse osmosis, and UV-hydrogen peroxide advanced oxidation
  - The permit considered supplementing wastewater with reclaimed stormwater and other impaired sources during dry spells to increase inflow reliability
- The utility (regional sanitation agency) operated as a joint-powers agency, which helped coordinate source control, treatment, conveyance, and injection across jurisdictions
- The state's groundwater replenishment framework dictated many aspects of the treatment process and application requirements on top of the river constraints, meaning this project operated under high regulatory stringency
- The project operated under a regulator-imposed cap on delivered cost, which created the need for a mix of external funding sources, including SRF and state/federal grants
  - Regulators had invited the utility to apply for a low-cost federal loan, an indicator of federal interest in the project
  - Operational costs paid by local agencies, including the wastewater utility implementing the project
- A demonstration facility served as the primary venue for public education, including tours, water-tasting, and a museum-style hall walking visitors through the treatment processes. Outside of the facility, school curriculum, plant tours, and community events characterized public awareness
  - This facility also doubled as an operator classroom, where AWTOs gained hands-on experience. Pay was tied to certification tiers
- Interagency collaboration was paramount, not only for managing multi-municipal involvement, but also in partnering with the regional water supply entity on compliance and delivery volumes

Case 10

Implementation outcome: Attempted

Driver: Early 2000s drought forced emergency water hauling

Method: Convert preexisting wastewater plant to an MBR (membrane bioreactor), gravity-feed permeate to an advanced treatment site (RO, UV-AOP), hold purified H2O in covered storage, blend with natural water sources, retreat through ultrafiltration (UF), UV, and (GAC)

Results: Portions of collection and conveyance were built, but project remained in exploratory phase

Details:

- Drought and emergency water hauling incentivized high-altitude village to invest in a water reuse project
- Project proposal was straightforward and ambitious for small scale
- Historically, potable reuse projects were permitted on a case-by-case basis for lack of specific regulations
- Case dominated by execution risk and infrastructure readiness constraints: contractor failures, degraded components, and unresolved design decisions delayed progress
    - Later, project installation was denied for lack of TMF capacity
- Contractor failure, faulty concrete and electrical work, components left to weather, and indecision on critical design element (brine concentration management) delayed project repeatedly
- Funding was external (grants and loans, intentioned at making construction free for residents), but was intermittent and frequently insufficient, no determined O&M funding (besides user fees), tiny base was unable to continually fund this high-value project
- Public interaction was mostly limited to informational meetings
    - Community conflicted over project, champions citing only source of “wet water” while critics continually used “toilet-to-tap" to describe the water
- Operators proactively pursued certifications (AMTA-affiliated MOC, out-of-state school training on RO/UF/MBR capacity) but staffing was thin

Case 11

Implementation outcome: Attempted

Driver: Utility's surface raw water source was vulnerable to drought, still had to meet flow constraints on nearby river systems, needed to supplement supplies during peak demand

Method: Many phases – initial feasibility program (augment raw water sources via natural treatment and subsurface storage), then "all options on the table" (including IPR, potential DPR, expansion of nonpotable distribution, and environmental flows benefits)

Results: Project was shelved and never broke ground

Details:

- Primary raw water source subject to drought (surface storage), not able to handle peak draw down
- Reuse seen as a cost-effective way to solve the resource problem and address a new statue that required utilities to eliminate non-beneficial surface discharges
- Regulation was very new, fragmented, and evolving, but nonetheless legitimized potable reuse
  - While technical feasibility work was substantial, the project was stranded by process and governance setbacks
- Utility had history with project planning that included studies and testing which progressed to a design-build concept. This time, lack of early public outreach yanked governing support before construction broke ground
- Project was planned to be funded predominantly with external funds, but no loans or federal funds were earmarked
- O&M planned to be funded entirely by rate revenue/user fees, later to be absorbed into the retail enterprise
- Utility assumed it was better to "have answers" before engaging the public, but that ultimately backfired. The feasibility and conceptual designs seemed to be foregone conclusions to stakeholders, causing them to feel excluded from decision-making
- No pilot for visitation limited public understanding of the need and benefits of reuse
- Project failed on process legitimacy and timing, not treatment performance

Case 12

Implementation outcome: Attempted

Driver: Maintain nitrogen reductions to remain in compliance with local estuary standards, stabilize a stressed coastal aquifer at risk of subsidence, needed to preserve groundwater resiliency in the face of growth and climate stressors

Method: MAR alongside other management layers, pilot (coag/floc → ozone/AOP → biofiltration/GAC → UV)

Results: ~0.5 MGD advanced treatment pilot, produced supporting data that carbon-based AWT met potable targets while remaining aquifer-compatible

Details:

- Compliance with regulations and a need for long-term resiliency initiated this project
- A statewide pilot statute suggested IPR pilot programs with specifications (environmental-buffer residence time, required RO and UV at the potable treatment stage, pathogen removal targets (12-10-10), explicit PFAS controls, plus public-participation provisions)
- Permitting for large-scale implementation of injections (different from IPR surface augmentation) was lacking, proved to be a crippling blow
- ~ 0.5 MGD pilot was built, followed the would-be largescale project process (pending permits), heavily monitored (ISAP)
- Utility had staged roadmap for "eventual" full-scale project implementation, and produced supporting data that carbon-based AWT met potable targets AND remained aquifer-compatible
- Any financing earmarked before project delay was entirely internal, external funds would be reserved for larger projects
- O&M funded through user fees, operator training was intended to be permit-led (once permits were issued...)
- Outreach focused on "people in power" (technical peers, NGOs, elected officials) and pilot tours were limited to invitation-only, making "outreach" more consultative than public-informing
- Interagency collaboration was strong, and collaboration was apparent in the utility's legislative posture
- Planning and credible data anchored the project, but permitting gap for large-scale injection and scaling workforce needs delayed the project

Case 13

Implementation outcome: Attempted

Driver: Needed to keep pace with rapid growth & seasonal low-flow constraints on local water sources

Method: First implemented a purification pilot to test a multibarrier treatment train, then evaluated two potable reuse pathways (indirect augmentation of raw-water reservoir, targeted base-flow support for the receiving stream through IPR)

Results: Water purification pilot w/goals of raw-water augmentation and ecological flow support, large data sets from pilot trials

Details:

- Water purification pilot was implemented to show that potable reuse was possible and effective (2 approaches evaluated)
- Extensive monitoring and "research-first" characterized the posture of the pilot, which ran through full trains and generated large data sets
- The state had nonpotable reuse rules already, but potable reuse rules and permitting were still being created (the utility was not included in the taskforce creating these rules)
- Planning was centered around raw-water augmentation and ecological flow support in the long term, per the utility's integrated management plan
- The pilot was half funded through local investments, and later secured a federal grant ($5.6million) toward an expanded "clean water" facility
    - The balance of capital was planned to be financed through state revolving fund loans and city funds
    - Rate planning updated periodically to maintain sufficiency
- The utility built a branded web presence, created a virtual tour of the pilot, and published regular updates, which familiarized potable reuse with the public. The public was involved in decision-making through briefings, open information channels, and formal comment opportunities through the state. Overall, public involvement was a strength
- Operators were trained through the pilot (which universities and staff ran and monitored), with future O&M funding planned to be absorbed into the enterprise
- Key permitting details remained unresolved, holding the project back, and the utility was not integrated into the rulemaking taskforce, creating uncertainty that constrained capital commitment and scaling timelines even as public involvement was generally a strength
    - Major capital commitments and pilot scaling was dependent on this

Case 14

Implementation outcome: Attempted

Driver: Needed to shift towards local water resource resiliency and seismic protections

Method: 50 MGD IPR plant with a potential to be sized to +200 MGD when fully built (wanted to preserve the possibility of a DPR transition), advanced treatment through membrane-based train with UV-AOP (state mandated), wastewater and drinking water utility collaboration

Results: Successful early public engagement but more than 30 year's delay in full-scale implementation

Details:

- Climate- and seismic-resiliency defined the long-term goals of the project, with "homegrown" water resources as the solution (as opposed to imported water)
- Drinking water utility and wastewater utility worked together under a "two-utility" cost-share philosophy and navigated a wastewater- and drinking water-regulator dual oversight structure
- Initial phase was planned to be 50 MGD with the possibility to expand to 200+ MGD
- Political clock (2030) set by local supply targets and a pledge to recycle all wastewater
- Conveyance build-out from the major water reclamation hub to receiving systems was a critical path barrier, preventing any large production
- The project was primarily local enterprise-funded (e.g. bonds, rate revenues) in the initiation phase, and no record of large federal credit for future implementations (no funding sources, internal or external, had been earmarked for the project)
- Economic reliability valued more than cheapest unit-water (found through economic resilience model)
- O&M planned to be absorbed into the enterprise, but staffing for operations was lacking
    - Operators would need to be qualified for DPR (eventually)
- Public education and awareness programs were formal pillars, and involved a dedicated program site, community meetings, and continuous engagement, especially with communities historically affected by infrastructure siting
- Interagency collaboration was necessary and was gradually proving to be a strength
- The city was involved throughout, from mayoral policy targets fueling initiation, to vetting the project's governance of operations through city resilience leadership for a
- This case demonstrated that early public engagement can be strong while other pathway conditions remain binding: conveyance build-out and infrastructure integration were critical path barriers, limiting near-term production despite ambitious scaling goals
    - Further, while early-phase financing relied largely on local enterprise funds, the record noted that large future funding sources were not earmarked and staffing for operations was still lacking

Case 15

Implementation outcome: Attempted

Driver: Supply stress on a tri-city, shared water reservoir created a need for additional supply sources for supply reliability

Method: Raw water purchase as a first option, wells as a second option, and IPR saved as a third

Results: Delayed till historical interagency commitments get resolved

Details:

- A reservoir shared by three cities was vulnerable to supply stress and contamination (SWS designated), causing inter-city disputes in dry years
- The state maintained an IPR chapter with a 6-step permitting path: feasibility, (if needed) pilot protocol, waste load allocation and modeling, engineering report, permit application with contingency plan, and final design
  - Additionally for SWS projects, must meet anti-degradation and assimilative-capacity limits, explicit pathogen and performance benchmarks (e.g., 5-log adenovirus, 3-log Crypto/Giardia) and robust O&M, reporting, and monitoring requirements
- A program had been developed and tested at a field pilot run by the existing reclamation facility, but infrastructure readiness for full-scale built implementation was lacking
  - Pilot evaluated a multi-step advanced treatment process, and samples from the pilot showed low effluent turbidity, frequent non-detects or single-digit pathogen counts post-UV, strong nitrogen and phosphorus polishing, and consistent micropollutant degradation
  - Pilot was run by utility staff, enabling operator training before any full-scale commissioning (dual Class A water and wastewater certifications were required by the state for key IPR operators, planned into workforce pathway)
- City viewed reuse as a tool in a larger scheme to ensure reliability and water quality instead of the main solution
- Financing involved a blend of local revenue bonds and SRF, where future funds would come entirely from the utility
- Public education and awareness were high, fueled by publicly reported encouraging pilot results and tours/tastings
- Decision-making participation was also high but proved detrimental as both other cities expressed concerns over cost-sharing agreements.
- The project was delayed over SWS designation and the complexity of multi-party governance and decision-making
- Delay driven by multi-party governance complexity and cost-sharing conflict across cities sharing a reservoir

Case 16

Implementation outcome: Attempted

Driver: Environmental and resource stewardship to plan for future population growth and protect the sensitive river system nearby

Method: DPR through MF/UF → ozone → biologically active carbon → UV-AOP

Results: Effluent sampling was ongoing but no really impetus for the project

Details:

- DPR framed as a method for resilience and stewardship -- preventing future need (due to population stress) and protecting natural resources (a sensitive river system), DPR one of many options to meet these needs (including conservation, aquifer recharge)
- Project was federally engineered with the following phases: effluent sampling, bench/pilot testing of parallel advanced-treatment trains (~250 GPM twin skids), environmental review, then final design (project remains in the sampling phase)
  - Supported in two regional reclamation offices
  - State Advanced Water Purification (AWP) program formulated a technical advisory group, finalized rule updates to expand potable reuse path, and introduced a dedicated Advanced Water Treatment (AWT) operator certification
- Water quality dependencies and lab logistics (availability and scope of labs) were a critical infrastructure path that prevented transition to the next project phase
- Early projects funded through federal science/technology grants and local enterprise funds, full-scale implementation funds hadn't been earmarked (planned as federal loans)
  - Estimate for the DPR facility landed near $25–35M, needed value engineering to keep user rate impacts moderate
- Staffing and AWT operator certification were binding constraints, diminishing workforce added to that pressure, year-long pilot was designed to relieve the pressure through training of operators in partnership with university programs
- Major public outreach was deferred until pilot water was available, planned a VR plant tour and small-group sessions at senior centers, earlier K-8 programs planned to be revitalized with the pilot
  - Cooperation with neighboring communities (and, where relevant, tribal governments) on shared water challenges was necessary to align with the project's collaboration demands
- The case had not yet accumulated the full bundle of institutional capacity conditions (e.g., mature operator training systems, committed multi-agency agreements, durable financing commitments, and sustained positive framing)

References

1. Yin RK. The Case Study Method as a Tool for Doing Evaluation. *Current Sociology*. 1992;40(1):121-137. doi:10.1177/001139292040001009

2. Sardana P, Javernick-Will A, Cook SM. Facilitators and Barriers of Global Water Reuse: A Systematic Literature Review. *ACS EST Water*. Published online December 26, 2024. doi:10.1021/acsestwater.4c00778

3. Pugel K, Javernick-Will A, Peabody S, et al. Pathways for collaboratively strengthening water and sanitation systems. *Science of The Total Environment*. 2022;802:149854. doi:10.1016/j.scitotenv.2021.149854

4. Schneider CQ, Wagemann C. *Set-Theoretic Methods for the Social Sciences: A Guide to Qualitative Comparative Analysis*. Cambridge University Press.; 2012.

5. Scruggs CE, Hacker ME. A Review of Social and Organizational Barriers to Water Reuse in the United States. *WIREs Water*. 2025;12(1):e70009. doi:10.1002/wat2.70009

6. Kyungsun Lee, Wendy Jepson. Drivers and barriers to urban water reuse: A systematic review. *Water Security*. 2020;11. doi:https://doi.org/10.1016/j.wasec.2020.100073

7. US EPA O. National Environmental Policy Act. July 19, 2013. Accessed November 5, 2025. https://www.epa.gov/nepa

8. fs/QCA Software. Accessed October 7, 2025. https://sites.socsci.uci.edu/~cragin/fsQCA/software.shtml

9. Ragin C. User's Guide to Fuzzy-Set / Qualitative Comparative Analysis. Published online July 2017. https://sites.socsci.uci.edu/~cragin/fsQCA/download/fsQCAManual.pdf

10. Ragin CC. *Redesigning Social Inquiry: Fuzzy Sets and Beyond*. University of Chicago Press; 2008.
